# Comparative Genomics of Environmental and Clinical *Stenotrophomonas maltophilia* Strains with Different Antibiotic Resistance Profiles

**DOI:** 10.1093/gbe/evv161

**Published:** 2015-09-14

**Authors:** Benjamin Youenou, Sabine Favre-Bonté, Josselin Bodilis, Elisabeth Brothier, Audrey Dubost, Daniel Muller, Sylvie Nazaret

**Affiliations:** ^1^Université de Lyon, France; Research Group on Environmental Multi-Resistance and Efflux Pump, CNRS, Ecole Nationale Vétérinaire de Lyon, and Université Lyon 1, UMR 5557 Ecologie Microbienne, Villeurbanne, France; ^2^EA4312 Laboratoire de Microbiologie Signaux et Microenvironnement, Université de Rouen, Mont-Saint-Aignan, France

**Keywords:** *Stenotrophomonas*, antibiotic resistance, phylogeny, efflux pump

## Abstract

*Stenotrophomonas maltophilia*, a ubiquitous Gram-negative γ-proteobacterium, has emerged as an important opportunistic pathogen responsible for nosocomial infections. A major characteristic of clinical isolates is their high intrinsic or acquired antibiotic resistance level. The aim of this study was to decipher the genetic determinism of antibiotic resistance among strains from different origins (i.e., natural environment and clinical origin) showing various antibiotic resistance profiles. To this purpose, we selected three strains isolated from soil collected in France or Burkina Faso that showed contrasting antibiotic resistance profiles. After whole-genome sequencing, the phylogenetic relationships of these 3 strains and 11 strains with available genome sequences were determined. Results showed that a strain’s phylogeny did not match their origin or antibiotic resistance profiles. Numerous antibiotic resistance coding genes and efflux pump operons were revealed by the genome analysis, with 57% of the identified genes not previously described. No major variation in the antibiotic resistance gene content was observed between strains irrespective of their origin and antibiotic resistance profiles. Although environmental strains generally carry as many multidrug resistant (MDR) efflux pumps as clinical strains, the absence of resistance–nodulation–division (RND) pumps (i.e., SmeABC) previously described to be specific to *S. maltophilia* was revealed in two environmental strains (BurA1 and PierC1). Furthermore the genome analysis of the environmental MDR strain BurA1 showed the absence of SmeABC but the presence of another putative MDR RND efflux pump, named EbyCAB on a genomic island probably acquired through horizontal gene transfer.

## Introduction

*Stenotrophomonas maltophilia* is an aerobic, nonfermentative Gram-negative bacillus belonging to the gamma class of the proteobacteria (Denton and Kerr 1998). This ubiquitous bacterium can be found in various environments, such as water, soil, rhizosphere, plants, food, and hospital settings among others (Brooke 2012). In the soil and especially in the rhizosphere that are known to be its main habitats, *S. maltophilia* can engage in beneficial interactions with plants by promoting their growth and protecting them against fungal and bacterial plant pathogens (Ryan et al. 2009). Some *S. maltophilia* strains are also known for their biotechnological potentials as they can contribute to bioremediation and phytoremediation strategies (Antonioli et al. 2007; Pages et al. 2008) and to the production of biomolecules of economic value (Ryan et al. 2009). Nevertheless, in clinical environments *S. maltophilia* constitutes an emerging opportunistic pathogen responsible for a wide array of nosocomial infections, such as pneumonia, bloodstream and urinary tract infections, endocarditis, and meningitis among immunocompromised or debilitated patients as well as among patients with cystic fibrosis (Looney et al. 2009). Community-acquired infections are rare but documented (Falagas et al. 2009).

One of the major features of clinical isolates of *S. maltophilia* is their high resistance levels toward most of the currently used antimicrobial agents, including β-lactams, carbapenems, macrolides, cephalosporines, fluoroquinolones, aminoglycosides, chloramphenicol, tetracyclines, and polymixines (Brooke 2012). Moreover, emerging resistance against the current “treatment of choice” trimethoprim–sulfamethoxazol is increasingly being reported in clinical isolates (Al-Jasser 2006; Toleman et al. 2007). Thus, therapy against infections caused by multidrug resistant (MDR) *S. maltophilia* presents a significant challenge for both clinicians and microbiologists. In its main habitat, *S. maltophilia* usually presents lower levels of resistance to antibiotics than clinical strains. However, in some instances, MDR isolates have been isolated from soils and aqueous environments (Berg et al. 1999; Alouache et al. 2012). These MDR environmental strains may therefore constitute a public health risk.

*Stenotrophomonas maltophilia* display many intrinsic antibiotic resistance mechanisms such as low membrane permeability (Hancock 1998; Mett et al. 1988) and the presence of chromosomally encoded antibiotic modifying enzymes such as β-lactamases (Walsh et al. 1994, 1997; Avison et al. 2001) and other aminoglycoside phospho- and acetyl-transferases (Li et al. 2003; Okazaki and Avison 2007). But, like most other bacterial pathogens, the major intrinsic resistance mechanism responsible for its MDR phenotype can be attributed to the activity of chromosomally encoded multidrug efflux pumps (Zhang and Poole 2000; Blair and Piddock 2009). These pumps are capable of active extrusion of noxious compounds out of the cell and can be specific to a substrate or to a broad range of compounds (Nikaido and Pages 2012). They are distributed among six families: The Multidrug and Toxic compound Extrusion (MATE) family, the small multidrug resistance (SMR) family, the major facilitator superfamily (MFS), the ATP-binding cassette (ABC)-transporter family, the resistance–nodulation–division (RND) family (Li and Nikaido 2009), and the fusaric acid resistance efflux pump family that has recently been described (Hu et al. 2012).

Efflux pump encoding genes are present in all bacterial chromosomes (Martinez et al. 2009). In addition, efflux pumps provide resistance to many structurally different antibiotics, including quinolones, a family of synthetic antibiotics (Nikaido and Pages 2012). Therefore, antibiotic resistance is probably only a secondary (more recent) function of these pumps. Indeed, some efflux pumps are involved in bacterial virulence, plant–bacteria interactions, trafficking of the quorum-sensing molecules and, more generally, in detoxification of not only intermediate metabolites or toxic compounds such as heavy metals and solvents, but also antibiotics naturally produced by other microorganisms (Alvarez-Ortega et al. 2013). These initial (ecological) roles of the efflux pumps have been probably the main force responsible for their functional and structural diversity, as well as the spread of these efflux pumps through the whole bacterial domain. To understand the emergence of MDR phenotypes, it is important to focus on the roles and diversity of these efflux pumps in a nonclinical context, especially for opportunistic pathogens, which present particular predispositions to quickly develop new antibiotic resistances.

In *S. maltophilia*, the RND efflux pumps family and its involvement in MDR phenotypes are the most documented. The RND efflux systems generally form tripartite components composed of a periplasmic membrane fusion protein (MFP), an inner membrane RND transporter, and an outer membrane factor (OMF) (Li and Nikaido 2009). Eight RND efflux pumps, SmeABC, SmeDEF, SmeGH, SmeIJK, SmeMN, SmeOP-TolC, SmeVWX, and SmeYZ, have been identified in the first sequenced *S. maltophilia* genome (Crossman et al. 2008). Among them, SmeABC, SmeDEF, SmeIJK, SmeOP-TolC, SmeVWX, and SmeYZ have been experimentally characterized and confirmed as involved in MDR phenotypes (Alonso and Martinez 2001; Li et al. 2002; Crossman et al. 2008; Chen et al. 2011; Gould et al. 2013; Lin et al. 2014). Interestingly, a recent study has shown that the SmeIJK pump is probably involved also in cell envelope integrity maintenance, illustrating the multifunctionality of efflux pumps, including those involved in MDR phenotypes (Huang et al. 2014). Like in various other bacterial species and especially in Gram-negative bacteria, antibiotic resistance mechanisms can also be acquired by *S. maltophilia* through horizontal gene transfer (HGT) events associated with mobile DNA elements such as phages, integrons, transposons, and plasmids carrying antibiotic resistance genes (Avison et al. 2000; Liaw et al. 2010; Hu et al. 2011). Nevertheless, in a start-up comparison of the two first sequenced genomes of *S. maltophilia*, it appeared that most antibiotic resistance genes, and especially the efflux pump encoding genes, are not associated with mobile genetic elements (Ryan et al. 2009).

Whole-genome sequencing has become a powerful tool to address ecological questions in microbiology. The first *S. maltophilia* whole genome sequenced was that of the clinical MDR strain K279a isolated from a bloodstream infection (Crossman et al. 2008). The study of the genomic content of this strain revealed a wide array of antibiotic resistance genes including many efflux pumps. Shortly thereafter, the whole-genome sequence of the endophytic strain R551-3 isolated from the poplar *Populus trichocarpa* was completed and compared with genomic sequences of other endophytes with the aim to start deciphering the mechanisms that underlie promotion of plant growth (Taghavi et al. 2008). Since the completion of these two whole-genome sequences, 11 other strains from clinical and environmental origin have been fully sequenced.

Antibiotic resistance profiles and the genes responsible for these properties, including those encoding efflux pumps of the RND family are well documented in a clinical context. Nevertheless, little is known in the environmental context. In this study, we tried to fill this gap by sequencing the whole genomes of three soil originating strains with known antibiotic resistance profiles, two of them showing higher antibiotic resistance levels than reference clinical MDR strains. These genomic data combined with those available in the public archives have constituted a good database for the implementation of a comparative genomic survey of the antibiotic resistance determinants of *S. maltophilia* to try deciphering the origin of efflux pumps associated with MDR phenotypes among environmental strains of *S. maltophilia*. In this study, the phylogenetic relationships between our three strains and the 11 genomes available in National Center for Biotechnology Information (NCBI) were investigated and the antibiotic resistance gene contents of these genomes were compared and discussed with regards to a strain’s geographical origin and their antibiotic resistance phenotypes.

## Materials and Methods

### Bacterial Strains and Sampling Sites

Three strains of *S. maltophilia* from our team’s strain collection (table 1) were selected for genomic sequencing based on their particular antibiotic resistance profiles. Two of these strains (BurA1 and BurE1) were isolated, in the periphery of Ouagadougou, Burkina-Faso, from bulk soil samples collected in sorghum fields. The remaining strain (PierC1) was isolated from soil sampled from the Pierrelaye plain. This plain is heavily contaminated with heavy metals and antibiotics (Tamtam et al. 2011) as it was amended since the 1890s with raw wastewater from Paris, France. These three strains were isolated and identified as *S. maltophilia* as described by Pinot et al. (2011).

**Table 1:** Source and Location of the *Stenotrophomonas maltophilia* Strains Studied

*Stenotrophomonas maltophilia* Strain	Source	Location	Reference	GenBank Accession Number	Antibiotic resistance
Clinical origin					
Ab55555	Clinical			ALOG00000000	Unknown
AU12-09	Catheter tip	(Australia)	Zhang et al. (2013)	APIT00000000	Unknown^a^
D457	Clinical	Mostoles (Spain)	Lira et al. (2012)	NC_017671.1	TET, ERY, NAL, NOR, OFX
K279a	Blood infection	Bristol (UK)	Crossman et al. (2008)	NC_010943.1	Multi-drug^b^
S028	Sputum	Beijing (China)	Song et al. (2012)	ALYK00000000	Multi-drug
Environmental origin					
JV3	Rhizosphere	(Brazil)	Lucas et al. (2011)	NC_015947.1	Unknown
PML168	Rock pool	Wembury (UK)	Allen et al. (2012)	CAJH00000000	Unknown
R551-3	Poplar tree endophyte	Washington state (USA)	Taghavi et al. (2008)	NC_011071.1	Sensitive^b^
RR10	Rice root	Zhejiang Province (China)	Zhu et al. (2012)	AGRB00000000	Unknown^a^
SKA14	Sea water	Baltic sea (Norway)	Hagström et al. (2013)	ACDV00000000	Unknown
BurA1	Soil	Ouagadougou (Burkina Faso)	This study		Multi-drug^b^
BurE1	Soil	Ouagadougou (Burkina Faso)	This study		Multi-drug^b^
PierC1	Soil	Pierrelaye (France)	This study		Sensitive^b^
Other origin					
EPM1	*Giardia duodenalis* culture contaminant	(Portugal)	Sassera et al. (2013)	AMXM00000000	Multi-drug^a^

Note.—TET, tetracycline; ERY, erythromycin; NAL, nalidixic acid; NOR, norfloxacin; OFX, ofloxacin.

^a^Strains for which the antibiotic resistance profile is not described in the references but presence of numerous antibiotic resistance genes are mentioned.

^b^Strains for which the antibiotic resistance profiles were evaluated in the present study.

Eleven previously sequenced strains from environmental (*n* = 5), clinical (*n* = 5), and other (*n* = 1) origins were included in the genomic analysis (table 1). The genome sequences were obtained from the NCBI (http://www.ncbi.nlm.nih.gov/) and are referenced with the accession numbers showed in the table 1. The clinical reference strain K279a (Avison et al. 2001) and the environmental reference strain R551-3 (Taghavi et al. 2008) were kindly provided by Dr Matthew B. Avison and Dr Daniel van der Lelie, respectively.

### Antibiotic Resistance Test

The in vitro antimicrobial resistances of the three newly sequenced *S. maltophilia* strains and the two reference strains K279a and R551-3 were determined using the Vitek2 system with a NO93 card dedicated to nonfermenting Gram-negative bacteria (bioMérieux, Marcy l’Etoile, France) according to manufacturer instructions. Minimal inhibitory concentrations (MIC) of 18 antibiotics were determined. MIC results were analyzed by the AESTM (Advanced Expert System) software incorporated in this system. The disk diffusion method was also used for strains BurA1 and BurE1. The phenotype for aminoside resistance was compared with that of the reference strains *Escherichia coli* ATCC 25 922 as request by the CA-SFM/EUCAST (Antibiogram Committee of the French Microbiology Society) and *S. maltophilia* K279a.

### Genome Sequencing, Assembly, and Annotation

Genomic DNA of the three sequenced strains was extracted from an overnight culture grown in TSB medium at 28 °C under agitation at 180 rpm. The genomic DNA extraction protocol was achieved as described previously (Pitcher et al. 1989).

BurA1 whole genome was sequenced using a Roche 454 GS Junior sequencer (454 Life Sciences, Branford, CT) combined with an Illumina Hiseq 2000 approach (Illumina, San Diego, CA). The 454 run was performed at the University of Lyon (France) by the DTAMB/Biofidal structure and led to 131,210 reads with an average read length of 423 bp. The 2 × 100 bp paired-end Hiseq run was performed by Genoscreen society (Lille, France) with a final number of 2 × 73,799,133 reads. The 454 reads were first de novo assembled using Newbler 2.6 (Roche) with an estimated average coverage of 13-fold. Hiseq reads were then mapped on the de novo assembly using BWA-MEM software (Li and Durbin 2009). The mapping of the Illumina reads raised the estimated coverage to 2,500-fold.

BurE1 and PierC1 whole genomes were sequenced at the University of Lyon (France) by the DTAMB/Biofidal structure using a Roche 454 GS Junior sequencer. For each strain, the number of reads was 186,793 and 160,103, respectively, with an average read lengths of 434 and 446 bp. The estimated average coverage was 18-fold and 15-fold, respectively.

For the three strains, the large contigs (size > 500 bp) were reordered relative to the genome sequence of the reference strain K279a using the Mauve Contig Mover (Rissman et al. 2009) of the MAUVE software (Darling et al. 2004). The contigs that could not be identified relative to the K279a genome sequence (one for each strain) were placed at the end of the alignment.

Coding sequences (CDSs) predictions, as well as automatic and manual sequence annotations, were performed using the MicroScope platform pipeline at Genoscope (Vallenet et al. 2013). Results are available through the MaGe graphical interface (Vallenet et al. 2006). CDSs were predicted using AMIGene software (Bocs et al. 2003). Automatic functional annotation of the predicted CDSs was performed using the tools integrated in the MicroScope platform (Vallenet et al. 2009) and the available annotations of the strain K279a and other related genomes. Gene predicted to be involved in functions of interest was manually checked by using the “genome browser” tool of the platform. Genomic islands and regions of genomic plasticity (RGPs) of each genome were identified using the “RGP finder” tool included in the MicroScope platform by comparing the genome of each query against all the other studied genomes as reference.

The nucleotide sequences of the strains BurA1, BurE1, and PierC1 were deposited into European Nucleotide Archive (http://www.ebi.ac.uk/ena) with the accession numbers ERS685922, ERS685923, and ERS685924, respectively (the study accession is PRJEB8824).

### Phylogenetic Analysis

The evolutionary relationships among the 14 studied *S. maltophilia* strains were determined from a concatenated alignment of the orthologous protein sequences of the core genome of these 14 strains. Orthologous proteins were identified from bidirectional best hit BLASTP searches of each strain proteome against K279a’s proteome with an *e*-value parameter threshold of 10e-5. Customized computer scripts were then used to extract the best reciprocal hits from all the strains and to align these protein sequences with Clustal omega (Sievers et al. 2011). The alignments were then filtered using Gblocks version 0.91 b (Talavera and Castresana 2007) with default options and concatenated. A final alignment of 1,647 concatenated proteins (514,787 amino acids) was used in the phylogenetic analyses. A phylogenetic tree was reconstructed with the maximum-likelihood method by implementation in RAxML V7.9.5 (Stamatakis 2006) with 1,000 bootstraps replicates. To root the phylogenetic tree, the same protocol was reiterated with the *Xanthomonas campestris* pv. *campestris* strain ATCC33913 genome as outgroup (Da Silva et al. 2002). In this case, a final alignment of 1,435 concatenated proteins (444,554 amino acids) was used in the analysis.

Additional phylogenetic studies were performed using different protein sequences (RND and integrase). In the same way as the phylogeny from orthologous proteins, the sequences were aligned with Clustal omega, then filtered using Gblocks. Phylogenetic tree was reconstructed with the maximum-likelihood method by implementation in RAxML V7.9.5 with 1,000 bootstraps replicates.

### Antibiotic Resistance Gene and Efflux Pumps Content Identification

Antibiotic resistance genes and efflux pumps were identified by keyword searches after automatic and manual annotation of the CDS. Searches were also performed using InterPro database family identifier numbers (Hunter et al. 2009). InterPro IDs were attributed to CDS by the InterProScan software (Quevillon et al. 2005) during the annotation process. Putative resistance and/or efflux functions were confirmed using BLASTP against the nonredundant protein sequence database. Known antibiotic resistance genes and efflux pumps described in *S. maltophilia* but not retrieved by the two previous methods were searched in the draft genomes by BLASTP searches after retrieving these sequences in the GenBanK database (Benson et al. 2013). We used an *e*-value parameter higher than 100 to confirm that no partial gene sequences were positioned at a contig extremity.

## Results

### Antibiotic Susceptibility Profiles

MICs across 18 antibiotics and combinations were tested using the Vitek2 system. *Stenotrophomonas maltophilia* strains showed differential susceptibilities as K279a, BurA1 and BurE1 showed low antibiotic susceptibility, whereas R551-3 showed intermediate susceptibility and PierC1 showed high susceptibility (table 2). Intermediate levels of resistance were considered as effective resistances. The two environmental MDR strains BurA1 and BurE1 showed increased resistances compared with strain K279a, the antibiotic resistant strain of reference. In contrast, the PierC1 strain showed a more sensitive phenotype than R551-3 that showed a medium resistance level. Strains BurA1 and BurE1 showed in vitro resistance to almost all the antibiotic classes assayed (resistance to 15 and 12 antibiotics over 18, respectively). These resistances encompass penicillins, cephalosporins, monobactam, carbapenems including meropenem, aminoglycosides, and polymixin. Although BurA1 is resistant to fluoroquinolones, all other strains except K279a were found to be sensitive. Reference clinical MDR strain K279a showed in vitro resistance to penicillins, carbapenems, aminoglycosides excepted isepamycin, fluoroquinolones and polymixin, but not to cephalosporins. Nevertheless, the MICs observed for penicillins, aminoglycosides, and polymixins classes of antibiotics were lower than those observed for the two environmental MDR strains. In contrast, PierC1 is sensitive to almost all antibiotics except imipenem from the carbapenems class of antibiotics. The strain R551-3 showed resistance toward the two carbapenems assayed, penicillins, cefepim from the cephalosporins class of antibiotics but not to ceftazidime. All strains were sensitive to ticarcillin from the penicillins class of antibiotics in combination with clavulanic acid and to minocycline from the tetracyclines class of antibiotics.

**Table 2:** Antibiotic MIC Profiles and Resistance Interpretation of *Stenotrophomonas maltophilia* Strains BurA1, BurE1, PierC1, K279a, and R551-3

Drug	Class	BurA1		BurE1		PierC1		K279a		R551-3	
		MIC (µg/ml)	Interpretation	MIC (µg/ml)	Interpretation	MIC (µg/ml)	Interpretation	MIC (µg/ml)	Interpretation	MIC (µg/ml)	Interpretation
Ticarcillin	Carboxypenicillin	≥128	R	≥128	R	≤8	S	64	R	≥128	R
Ticarcilline/ clavulanic acid	Combination	ND	ND	≤8	S	≤8	S	≤8	S	≤8	S
Piperacillin	Ureidopenicillin	64	R	≥128	R	16	S	32	I	≥128	R
Piperacillin/ tazobactam	Combination	64	R	32	I	8	S	ND	ND	64	R
Ceftazidime	Cephalosporin	≥64	R	16	R	≤1	S	2	S	4	S
Cefepime	Cephalosporin	32	R	32	R	≤1	S	4	S	16	R
Aztreonam	Monobactam	≥64	R	≥64	R	ND	ND	≥64	R	≥64	R
Imipenem	Carbapenem	≥16	R	≥16	R	≥16	R	≥16	R	≥16	R
Meropenem	Carbapenem	≥16	R	≥16	R	≤0.25	S	≥16	R	≥16	R
Amikacin	Aminoglycoside	≥64	R	≥64	R	≤2	S	16	R	4	S
Gentamicin	Aminoglycoside	≥16	R	≥16	R	≤1	S	8	R	≤1	S
Isepamycin	Aminoglycoside	≥64	R	≥64	R	2	S	8	S	8	S
Tobramycin	Aminoglycoside	8	R	≥16	R	≤1	S	≥16	R	≤1	S
Ciprofloxacin	Fluoroquinolone	2	R	1	S	0.5	S	2	R	0.5	S
Pefloxacin	Fluoroquinolone	2	I	1	S	1	S	2	I	0.5	S
Minocycline	Tetracycline	≤1	S	≤1	S	≤1	S	≤1	S	≤1	S
Colistin	Polymyxin	≥16	R	≥16	R	≤0.5	S	8	R	≤0.5	S
Trimethoprim/ sulfamethoxazole	Sulfonamide	40	S	≤20	S	≤20	S	≤20	S	≤20	S

Note.—Interpretations were made according to the recommendations of the antibiogram committee of the French society of microbiology. R, resistant; I, intermediate; S, sensitive. Intermediate interpretation was considered as resistant due to a health precautionary principle. ND, not determined.

### General Features of the Sequenced Genomes

The general genomic features of the three novel *S. maltophilia* environmental strains sequenced in this study are summarized in the table 3. The draft genomes of BurA1, BurE1 and PierC1 consisted of approximately 4,366,960, 4,509,290 and 4,644,375 bp circular chromosomes assembled in 64, 48, and 59 contigs of size greater than 500 bp, respectively. No plasmids were detected in the genome assemblies or by Pulse Field Gel Electrophoresis (PFGE) (data not shown). The average G+C % of BurA1 and BurE1 was 66.6% and this of PierC1 was 66.3%. These data are consistent with the average G+C % of the other *S. maltophilia* genomes (table 3). In total, 4,132, 4,223 and 4,422 predicted protein-CDSs were identified in the genomes of BurA1, BurE1 and PierC1, respectively, with an average CDS length of 952–962 bp and a coding density of 89.5–89.7%. These general CDSs features are also consistent with the CDSs features observed in the other strains (table 3). Sixty, 65 and 64 transfer RNA genes were, respectively, found among the genomes of BurA1, BurE1 and PierC1, which is within the range of what is observed among the other genomes. The ribosomal RNA operons copy number could not be determined because the three genomes were not finished and all the reads corresponding to these genomic regions were aligned and assembled in one operon by the assembling software. Between 91 and 101 RGPs were identified among each genome.

**Table 3:** General Genomic Features of the *Stenotrophomonas maltophilia* Strains Obtained from the MicroScope Annotation Platform and from the GenBank Platform

	Strains													
	Ab55555^a^	AU12-09^a^	D457^a^	EPM1^a^	K279a^a^	S028^a^	BurA1^b^	BurE1^b^	PierC1^b^	JV3^c^	PML168^c^	R551-3^c^	RR10^c^	SKA14^c^
Chromosome size (megabase pairs)	4.9	4.55	4.77	4.79	4.85	3.75	4.36	4.5	4.64	4.54	4.4	4.57	4.68	5.02
Plasmid	No	No	No	No	No	No	No	No	No	No	No	No	No	No
G+C (%)	66.1	66.5	66.8	66.4	66.3	67.1	66.6	66.6	66.3	66.9	66.6	66.3	66.3	66.4
Protein-CDSs	4,739	4,004	4,599	4,591	4,760	3,686	4,132	4,223	4,422	4,222	4,228	4,170	4,508	4,788
Average CDS length (nt)	937	NA	930	945	934	949	954	962	952	972	957	989	936	954
Coding density (%)	89.1	NA	88.5	89.6	89.3	91	89.5	89.5	89.7	89.5	90.3	89.5	89.3	90.5
Ribosomal RNA operons	2	NA	4	4	4	NA	NA	NA	NA	4	3	4	NA	4
Transfer RNA genes	70	70	71	66	74	37	60	65	64	73	57	73	106	70
# scaffolds	6	4	1	1	1	1	1	1	1	1	1	1	1	3
# contigs	21	125	1	19	1	297	64	48	59	1	93	1	158	49
RGPs	94	NA	96	94	94	92	95	92	101	95	95	95	91	99

Note.—As the annotation process of certain publicly available genome sequences was repeated in the MicroScope annotation platform, general genomic features may slightly differ from those given in the NCBI portal.

^a^Clinical strains available in the public databases.

^b^Environmental strains of *S. maltophilia* sequenced during this study.

^c^Environmental strains available in the public databases.

### *Stenotrophomonas maltophilia* Core Genome and Phylogeny

**Fig. 1.—:**
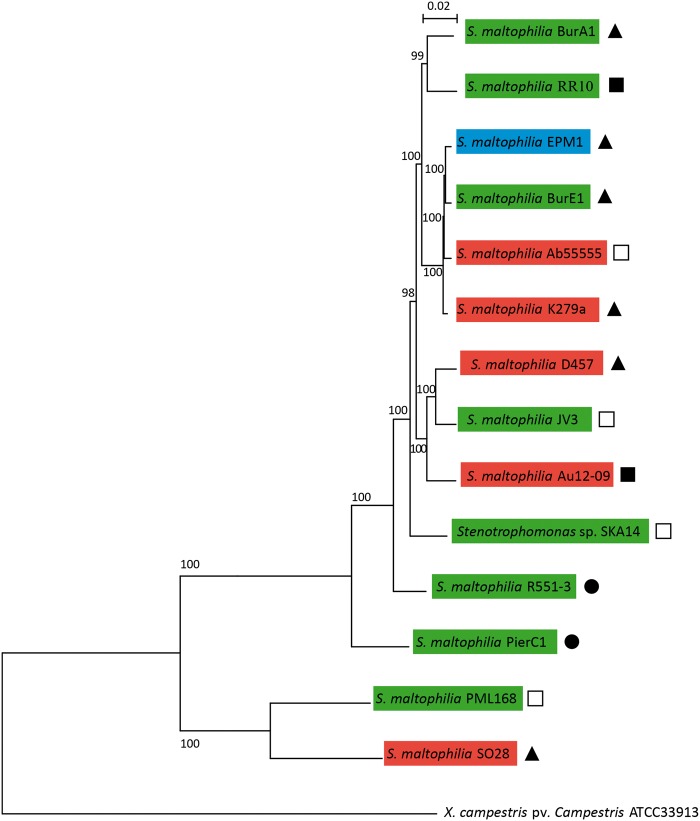
Phylogenetic tree from maximum-likelihood analysis of the core-genome alignments of the 14 strains of *S. maltophilia* and *X. campestris* pv *campestris* strain ATCC33913. In total, 1,435 orthologous proteins were concatenated in an alignment of 444,554 amino acids. Bootstraps are indicated at each node. Strains highlighted in green are from environmental origin, in red from clinical origin, and in blue from other origin. Strain names followed by a plain triangle are MDR; those followed by a plain circle are sensitive. Antibiotic resistance profiles of the strains followed by squares are unknown but presence of antibiotic resistance genes was related in the genome references of the strains followed by plain squares.

Using reciprocal BLASTP, protein-coding genes having a 1:1 orthologous relationship to each other were identified across the 14 available *S. maltophilia* genomes. A total of 1,647 CDSs were identified which could be considered the core set of orthologous genes, at least for those 14 strains. To root the phylogenetic tree, the core genome analysis was performed with a *Xanthomonas campestris* pv. *campestris* strain as outgroup. All the *S. maltophilia* and the *Xanthomonas* strains shared 1,435 orthologous proteins concatenated in an alignment of 444,554 amino acids used for phylogenetic tree reconstruction by maximum-likelihood method (fig. 1). Most of strains not grouped within clusters are from environmental origin, and the different clades revealed by the phylogenetic analysis are consistent neither with the sampling origin of the strains nor with their antibiotic resistance phenotypic properties (fig. 1). Indeed, clusters including both clinical and environmental strains have been revealed by the phylogeny. As an example, strain BurE1, isolated from Burkinabe soils, clustered with K279a and Ab55555 from clinical origin and EPM1, which is a laboratory culture contaminant. Moreover, the two clinical strains, D457 from Spain and AU12-09 from Australia, clustered with strain JV3 which was isolated from a rhizosphere sample from Brazil. This confirms that the core genome phylogeny does not allow the clustering of the strains according to their geographical origin and/or their habitat (i.e., environmental vs. clinical strains). In the same way, this phylogeny does not permit the discrimination of the MDR and antibiotic sensitive strains. Despite the lack of information for many sequenced strains, sensitive and resistant strains seem to group within different clusters (fig. 1). The antibiotic sensitive strains PierC1 and R551-3 that show intermediate levels of resistance are not grouped with other strains. Remarkably, the environmental MDR strain BurE1 is genetically close to three strains, including two MDR strains, K279a and EPM1. Unfortunately, the antibiotic resistance profile of the fourth strain of this clade, Ab55555, remains unknown. The environmental MDR strain BurA1 forms a clade with the strain RR10, which was isolated from a rice plant rhizosphere and is not related to antibiotic resistant strains, even if the presence of many antibiotic resistance genes in its genome was previously reported (Zhu et al. 2012).

### Overview of Antibiotic Resistance Genes

Antibiotic resistance genes were sought primarily in the genomes of the environmental strains sequenced in this study as well as in those of the reference strains K279a and R551-3 for which accurate data on their drug resistance profiles were available. On the basis of the core-genome phylogenetic clustering of the sequenced strains, other publicly available sequenced strains such as Ab55555, which clustered with strains BurE1 and K279a, and strain RR10, phylogenetically close to the strain BurA1, were added in the analysis. Strains D457 and JV3 grouped together and were included in the survey.

**Fig. 2.—:**
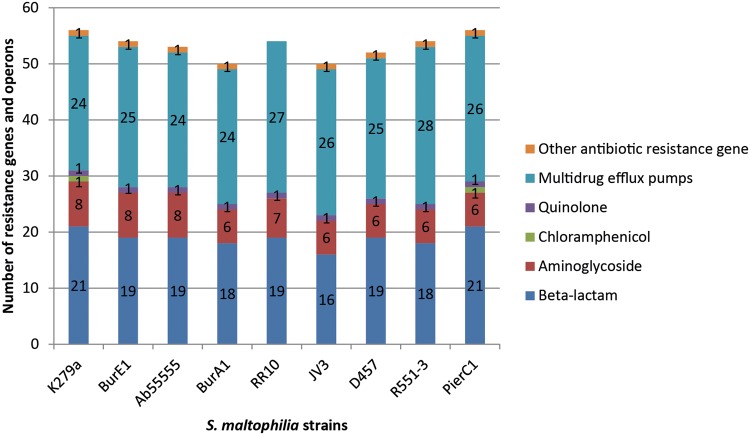
Summary of the antibiotic resistance genes and operons present in each *S. maltophilia* strains. Multidrug efflux pumps operons are counted as one even if encoded by multiple genes. No tetracycline and sulfonamide resistance genes were found.

Between 50 and 56 known or putative antibiotic resistance genes and efflux pumps commonly considered to be implied in MDR phenotypes were found among the nine strains, including genes involved in resistance to beta-lactam compounds such as penicillins and carbapenems, as well as aminoglycosides and quinolones (fig. 2). The number of identified genes cannot be linked with the resistance profiles observed. Indeed, the same number of resistance genes (*n* = 56) was found in the sensitive strain PierC1 and in the multiresistant strain K279a. Similarly, the intermediate resistant strain R551-3 and the multiresistant strain BurE1 share the same number of resistance genes (*n* = 54). Moreover, the lowest amount of resistance genes (*n* = 50) was found in the strain BurA1, which showed the highest resistance level among the studied strains.

Three known beta-lactamase encoding genes are shared by all the strains: *bla*L1 encoding a metallo-beta-lactamase, which is suspected to confer resistance to imipenem as well as *bla*L2 and *amp*C, which are cephalosporinase-like enzymes (table 4). Between 13 and 18 putative beta-lactamase encoding genes were found in each strain, with 12 genes shared by all the strains. Nevertheless, the number of known or putative beta-lactam genes present in each genome does not allow the distinction of the resistance phenotype of the different strains against this class of antibiotic as beta-lactam resistant strains do not carry an increased number of genes encoding beta-lactamase proteins compared with sensitive ones. For instance, the same number of putative beta-lactamase encoding genes has been detected among the sensitive strain PierC1 and the resistant strain K279a (*n* = 21) (fig. 2). These 2 genomes share 19 putative beta-lactamase encoding genes, 2 of them being unique to each genome (table 4). Moreover strains BurA1 and BurE1, which are resistant to almost all the tested antibiotics belonging to the beta-lactams, show a lower number of genetic determinants involved or putatively involved in resistance to beta-lactams. Similarly, the presence of the characterized cephalosporinases AmpC and BlaL2 in all the strains did not allow inference about their resistance profile for these antibiotics, PierC1 and K279a harboring these two genes but being sensitive to cephalosporins.

**Table 4:** Summary of the Antibiotic Resistance Genes Found among the Nine Strains of *Stenotrophomonas maltophilia* and Their Related Locus Tag

	K279a	BurE1	Ab55555	BurA1	RR10	D457	JV3	R551-3	PierC1
Known β-lactam resistance genes									
ampC	Smlt_0115	SmBFE_10085	ALOG_10036	SMBUR_70127	AGRB_1350003	SMD_0070	BurJV3_0070	Smal_0071	SmPIER_10096
Metallo-beta-lactamase l1 (*bla*L1)	Smlt_2667	SmBFE_210322	ALOG_110253	SMBUR_60016	AGRB_830068	SMD_2343	BurJV3_2191	Smal_2146	SmPIER_360009
Beta-lactamase l2 (*bla*L2)	Smlt_3722	SmBFE_330045	ALOG_130919	SMBUR_310038	AGRB_900050	SMD_3327	BurJV3_3170	Smal_3136	SmPIER_500109
*Putative* β*-lactam resistance genes*									
Metallo-beta-lactamase family protein	Smlt_0347	SmBFE_30019	ALOG_20149	SMBUR_130078	AGRB_1310078	SMD_0282	BurJV3_0271	Smal_0244	SmPIER_20102
Putative beta-lactamase (PBP 4)	Smlt_0462	SmBFE_50045	ALOG_30046	SMBUR_300002	AGRB_310002	SMD_0391	BurJV3_0373	Smal_0343	SmPIER_60045
Putative beta-lactamase	Smlt_0523	SmBFE_70005	ALOG_30105	SMBUR_250009	AGRB_1060016	SMD_0441	BurJV3_0424	Smal_0402	SmPIER_60109
Putative metallo beta-lactamase family protein	Smlt_0580	SmBFE_80053	ALOG_30160	SMBUR_230020	AGRB_1080022	SMD_0495	BurJV3_0475	Smal_0456	SmPIER_60162
Putative metallo beta-lactamase family protein	Smlt_0581	SmBFE_80054	ALOG_30161	SMBUR_230019	AGRB_1080023	SMD_0496	BurJV3_0476	Smal_0457	SmPIER_60163
Putative beta-lactamase	Smlt_1470	SmBFE_180025	ALOG_60039	SMBUR_80088	AGRB_910017	SMD_1305	BurJV3_1223	Smal_1234	SmPIER_160175
Putative metallo-beta-lactamase superfamily protein	Smlt_1815	SmBFE_190085	ALOG_60526	SMBUR_190042	AGRB_1210006	SMD_1751	BurJV3_1601	Smal_1554	SmPIER_170243
Putative beta-lactamase	Smlt_3114	SmBFE_280113	ALOG_130332	SMBUR_30157	AGRB_840024	SMD_2694	BurJV3_2565	Smal_2553	SmPIER_440066
Putative beta-lactamase	Smlt_3495	SmBFE_300159	ALOG_130697	SMBUR_40170	AGRB_750011	SMD_3077	BurJV3_2956	Smal_2923	SmPIER_460297
Putative beta-lactamase	Smlt_3652	SmBFE_320074	ALOG_130847	SMBUR_360003	AGRB_1350018	SMD_3220	BurJV3_3098	Smal_3068	SmPIER_500018
Putative metallo-beta-lactamase superfamily protein	Smlt_3807	SmBFE_330125	ALOG_150056	SMBUR_200003	AGRB_1380014	SMD_3409	BurJV3_3251	Smal_3222	SmPIER_500189
Putative metallo-beta-lactamase superfamily protein	Smlt_3991	SmBFE_370133	ALOG_160174	SMBUR_90113	AGRB_550016 - 1330023	SMD_3590	BurJV3_3439	Smal_3401	SmPIER_500370
Putative beta-lactamase	Smlt_2514	No	No	SMBUR_60142	No	No	No	Smal_2001	SmPIER_520126
Putative penicillin-binding protein/beta-lactamase	Smlt_2563	SmBFE_210214	ALOG_110150	SMBUR_60096	AGRB_970014	SMD_2246	No	No	SmPIER_390020
Putative beta-lactamase AmpC protein	Smlt_2589	SmBFE_210238	ALOG_110176	No	No	No	No	No	SmPIER_340017
Putative beta-lactamase	Smlt_3132	No	No	No	AGRB_840044	SMD_2710	No	Smal_2573	No
Putative beta-lactamase	Smlt_4159	SmBFE_380035	ALOG_160338	SMBUR_10037	No	No	No	No	No
Putative beta-lactamase	Smlt_4211	SmBFE_380084	ALOG_160388	No	AGRB_1510036	SMD_3811	BurJV3_3660	No	SmPIER_530027
Beta-lactamase domain containing protein	No	No	No	No	No	No	No	No	SmPIER_180030
Putative beta-lactamase	No	No	No	No	No	No	No	No	SmPIER_460142
Putative beta-lactamase	No	No	No	No	No	No	No	Smal_3669	No
Beta-lactamase class C	No	No	No	No	No	SMD_2256	No	No	No
Known and putative aminoglycoside resistance genes									
Putative aminoglycoside phosphotransferase	Smlt_0191	SmBFE_20029	ALOG_20042	SMBUR_180020	AGRB_1220017	SMD_0160	BurJV3_0146	Smal_0151	SmPIER_10177
Putative aminoglycoside phosphotransferase	Smlt_1131	SmBFE_170042	ALOG_50153	SMBUR_100042	AGRB_790032	SMD_1054	BurJV3_0970	Smal_0976	SmPIER_120004
Putative aminoglycoside 3′-phosphotransferase	Smlt_2120	SmBFE_200047	ALOG_60817	SMBUR_50152	AGRB_1250024	SMD_1909	BurJV3_1756	Smal_1717	SmPIER_180077
Putative spectinomycin phosphotransferase	Smlt_2125	SmBFE_200051	ALOG_60821	SMBUR_50156	AGRB_1250028	SMD_1912	BurJV3_1759	Smal_1721	SmPIER_180080
Streptomycin 3″-phosphotransferase	Smlt_2336	SmBFE_210106	ALOG_110013	SMBUR_160072	AGRB_860077	SMD_2105	BurJV3_1980	Smal_1923	SmPIER_31001
Putative aminoglycoside 2′-*N*-acetyltransferase	Smlt_1669	SmBFE_180214	ALOG_60334	No	AGRB_830023	No	No	No	No
Aminoglycoside 6′-*N*-acetyltransferase (*aac(6')-iz*)	Smlt_3615	SmBFE_320037	ALOG_130811	No	No	No	No	No	No
Dimethyladenosine transferase (*ksgA*)	Smlt_0818	SmBFE_100026	ALOG_30377	SMBUR_20181	AGRB_1140003	SMD_0699	BurJV3_0680	Smal_0668	SmPIER_60395
Putative chloramphenicol resistance gene									
Putative chloramphenicol acetyltransferase (*cat*)	Smlt_0620	No	No	No	No	No	No	No	SmPIER_60202
Known fluoroquinolones resistance gene									
Putative fluoroquinolone resistance protein qnrB (*smqnr*)	Smlt_1071	SmBFE_160067	ALOG_50092	SMBUR_100101	AGRB_720043	SMD_0998	BurJV3_0908	Smal_0911	SmPIER_110039
Other antibiotic resistance gene									
Phosphomannomutase/phosphoglucomutase (*spg*M)	Smlt_0403	SmBFE_30067	ALOG_20199	SMBUR_390016	No	SMD_0323	BurJV3_0314	Smal_0286	SmPIER_30018

Aminoglycoside phosphotransferase and aminoglycoside acetyltransferase enzymes mediate resistance to aminoglycoside drug class. All the strains carry five genes encoding putative or characterized aminoglycoside phosphotransferase enzymes including streptomycin 3′-phosphotransferase and spectinomycin phosphotransferase (table 4). Two putative aminoglycoside acetyltransferases were found among the genomes but they were not present in each strain. A putative aminoglycoside 2′-*N*-acetyltransferase was found in strains K279a, BurE1, and Ab55555 belonging to the same phylogenetic cluster, as well as in strain RR10. The characterized aminoglycoside 6′-*N*-acetyltransferase gene *aac*(6′)-iz encoding an aminoglycoside modifying enzyme responsible for the resistance toward amikacin, netilmicin, sisomicin, and particularly tobramycin was found in strains K279a, BurE1, and Ab55555. The BurA1 strain did not carry aminoglycoside acetyltransferase genes although it showed the same aminoglycoside resistance levels than the BurE1 strain and even higher resistance level than the strain K279a. On the opposite with the same aminoglycoside resistance gene content than the strain BurA1, PierC1 showed a sensitive phenotype. Thus, aminoglycoside resistance in BurA1 could result from specific membrane permeability and efflux pump content. The dimethyladenosine transferase *ksg*A gene involved in resistance to kasugamycin was also found in all the genomes.

All the strains also carry the Sm*qnr* chromosomal quinolone resistance gene. Nevertheless, among the five strains for which the antibiotic resistance profiles are available, only BurA1 and K279a are resistant to both fluoroquinolones assayed.

All strains but RR10 share the phosphoglucomutase *spg*M gene associated with resistance to polymyxin B, polymixin E, nalidixic acid, gentamicin, vancomycin, ceftazidime, ticarcillin–clavulanic acid, and piperacillin–tazobactam (Liaw et al. 2010). *SpgM* is not formerly an antibiotic resistance determinant as it encodes a phosphoglucomutase enzyme associated with lipopolysaccharides (LPS) biosynthesis. Nevertheless, it was shown to be moderately involved in antimicrobial resistance and in virulence (McKay et al. 2003).

The putative chloramphenicol resistance gene *cat* was found in strains K279a and PierC1.

No resistance genes were found for the tetracycline and sulfonamide classes.

### Efflux Pumps Related to Drug Resistance

**Fig. 3.—:**
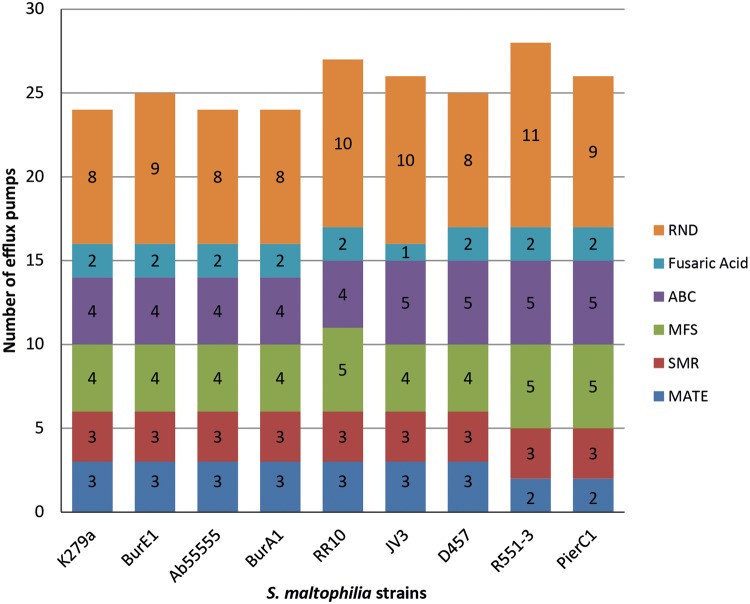
Summary of the known and putative multidrug efflux pumps found in the nine strains of *S. maltophilia*.

Given their prominent role in the antibiotic resistance in *S. maltophilia*, efflux pumps involved or putatively involved in drug-resistance were investigated in greater detail. Each of the six efflux pump families was explored for efflux systems involved in drug-resistance and antimicrobial-resistance. Efflux pumps related to metal compound resistance are not discussed here. In terms of presence/absence of genetic determinants encoding efflux pumps, little to no differences were observed between the nine genomes investigated (fig. 3). No correlation could be made between the number of putative and known MDR efflux pumps found in each genome and the antibiotic resistance phenotypes observed. As an example, the largest number of MDR efflux pumps (*n* = 28) was found in the environmental strain R551-3 that shows resistance toward fewer antibiotics than the MDR clinical strain K279a that harbors 24 MDR efflux systems (fig. 3). Similarly the extremely sensitive strain PierC1 harbors 26 MDR efflux systems likely to contribute to antibiotic resistance whereas BurA1 and BurE1, which are resistant to many antibiotics, carry 24 and 25 MDR efflux systems respectively.

### The MATE Family

MATE efflux pumps can export xenobiotic compounds like antibiotics of the quinolone class, antimicrobials and dyes, out of the bacterial cell. They are composed of a single transmembrane protein encoded by a single gene (Kuroda and Tsuchiya 2009). Three genes encoding MATE efflux pumps were found among the studied genomes, two of them being present in all of the strains (table 5). One of those encodes an efflux pump homologous to the characterized PmpM MATE efflux pump from *Pseudomonas aeruginosa* with translated amino acid sequence identity of about 40% over 99% of the protein sequence. The PmpM efflux pump confers resistance against ciprofloxacin, norfloxacin, ofloxacin, and against antimicrobials such as acriflavin and benzalkonium chloride. It is also known to extrude ethidium bromide out of the cell. The second gene encoding a MATE efflux pump found in all the strains possesses no characterized homolog. Nevertheless, its translated amino acid sequence shows conserved domains related to the NorM efflux pump described in *Vibrio parahaemolyticus* and *Escherichia coli*. Like PmpM and the other characterized MATE efflux pumps, the NorM efflux pump is known to confer resistance against quinolones and others antimicrobials. The third gene encoding a MATE efflux pump was found in all the strains excepted R551-3 and PierC1. Its translated amino acid sequence also shares conserved protein domains with the NorM efflux pump from *V. parahaemolyticus* and *E. coli*.

**Table 5:** Summary of the Efflux Pumps Involved or Putatively Involved in MDR Phenotype Found among the Nine Strains of *Stenotrophomonas maltophilia*

Family	Gene/homolog	K279a	BurE1		Ab55555		BurA1		RR10		JV3		D457		R551-3		PierC1		Substrate
			Locus	ID	Locus	ID	Locus	ID	Locus	ID	Locus	ID	Locus	ID	Locus	ID	Locus	ID	
MATE	*pmp*M	smlt1381	SmBFE_170227	98.8	ALOG_50360	99	SMBUR_80011	97.3	AGRB_900094	97.9	BurJV3_1146	97.3	SMD_1233	98.6	Smal_1160	96.7	SmPIER_160091	91.7	Ciprofloxacin, norfloxacin, ofloxacin, acriflavin, benzalkonium chloride, EtBr
		smlt2970	SmBFE_280022	93.4	ALOG_130224	95.1	SMBUR_30227	89.2	AGRB_1190043	92.7	BurJV3_2479	91.2	SMD_2608	90.8	No	No	No	No	[quinolones, antimicrobials, dyes]
	*nor*M	smlt4191	SmBFE_380064	99.6	ALOG_160367	99.8	SMBUR_10067	99.1	AGRB_1510014	98.7	BurJV3_3640	98	SMD_3790	97.8	Smal_3598	97.8	SmPIER_530005	97.1	
SMR	*sug*E	smlt1007	SmBFE_150007	100	ALOG_50022	100	SMBUR_20002	98.1	AGRB_430002	99.1	BurJV3_0859	99.1	SMD_946	98.1	Smal_0853	98.1	SmPIER_90108	98.1	CTAB, cetylpyridinium chloride, cetylpyridinium bromide, cetyldimethylethyl ammonium bromide
	*emr*E	smlt3363	SmBFE_300027	99.1	ALOG_130569	99.1	SMBUR_120032	98.2	AGRB_680070	92.3	BurJV3_2811	92.3	SMD_2935	93.6	Smal_2787	96.4	SmPIER_460115	90.9	Methyl viologen, tetraphenylphosphonium, benzalkonium, CTAB, cetylpyridinium chloride, EtBr, acriflavin/proflavin, crystal violet, pyronine, safranine, ampicillin, erythromycin, tetracycline
	*sug*E	smlt4304	SmBFE_380178	100	ALOG_160482	100	SMBUR_10170	99.1	AGRB_470010	100	BurJV3_3746	100	SMD_3898	99.1	Smal_3713	99.1	SmPIER_550005	96.2	CTAB, cetylpyridinium chloride, cetylpyridinium bromide and cetyldimethylethyl ammonium bromide
MFS	*emr*A	smlt1529	SmBFE_180083	100	ALOG_60096	100	SMBUR_80146	99.5	AGRB_730022	99	BurJV3_1279	98.7	SMD_1360	99.5	Smal_1288	98.5	SmPIER_160230	97.7	Carbonyl cyanide m-chlorophenylhydrazone, tetrachlorosalicylanilide, organomercurials, nalidixic acid, thiolactomycin
	*emr*B	smlt1530	SmBFE_180084	99.8	ALOG_60097	100	SMBUR_80147	99.2	AGRB_730023	99.1	BurJV3_1280	99.2	SMD_1361	100	Smal_1289	97.4	SmPIER_160231	97.5	
	OMF	smlt1528	SmBFE_180082	99	ALOG_60095	99	SMBUR_80145	97.2	AGRB_730021	98.6	BurJV3_1278	95.4	SMD_1359	97.6	Smal_1287	94.4	SmPIER_160229	92.4	
	*bcr*	smlt3578	SmBFE_320003	99.3	ALOG_130775	99.3	SMBUR_260025	98.8	AGRB_1340003	97.3	BurJV3_3029	98.1	SMD_3150	97.8	Smal_2999	96.8	SmPIER_480019	92.4	Bicyclomycin, sulfathiazole, chloramphenicol
	*mdt*D	smlt3623	SmBFE_320045	99.8	ALOG_130819	100	SMBUR_290027	99.4	AGRB_250008	99.6	BurJV3_3068	98.3	SMD_3192	98.7	Smal_3040	98.7	SmPIER_490021	96.4	Unknown
	OMF	smlt3969	SmBFE_370110	99.6	ALOG_160151	99.6	No	No	AGRB_360025	95.3	BurJV3_3416	97	SMD_3568	97.9	Smal_3378	97	SmPIER_500349	91.8	Carbonyl cyanide m-chlorophenylhydrazone, tetrachlorosalicylanilide, organomercurials, nalidixic acid, thiolactomycin
	*emr*A	smlt3970	SmBFE_370111	100	ALOG_160152	99.7	No	No	AGRB_360026	96.9	BurJV3_3417	95.6	SMD_3569	98.1	Smal_3379	99.4	SmPIER_500350	89.2	
	*emr*B	smlt3971	SmBFE_370112	99.8	ALOG_160153	99.8	No	No	AGRB_360027	99.2	BurJV3_3418	98.6	SMD_3570	99.6	Smal_3380	98.4	SmPIER_500351	94.8	
	*bcr*/*cfl*A	No	No	No	No	No	SMBUR_10029	81.5	AGRB_150016	83.9	No	No	No	No	Smal_3550		SmPIER_520113	76.4	Bicyclomycin, sulfathiazole, chloramphenicol
ABC	*smr*A	smlt1471	SmBFE_180026	99.8	ALOG_60040	99.7	SMBUR_80089	99.2	AGRB_910018	98.7	BurJV3_1224	99.8	SMD_1306	99.3	Smal_1235	97.9	SmPIER_160176	96.6	Ciprofloxacin; norfloxacin, ofloxacin, tetracycline, doxorubicin, dyes
	OMF	smlt1537	SmBFE_180090	99.8	ALOG_60104	99.6	SMBUR_110004	95	AGRB_730030	98.3	BurJV3_1286	95.8	SMD_1370	95.2	Smal_1296	95	SmPIER_160237	92	Macrolides
	*mac*B	smlt1538	SmBFE_180091	99.9	ALOG_60105	100	SMBUR_110005	98.5	AGRB_730031	98.8	BurJV3_1287	98.5	SMD_1371	98.2	Smal_1297	97.9	SmPIER_160238	94.2	
	*mac*A	smlt1539	SmBFE_180092	99.5	ALOG_60106	100	SMBUR_110006	96.4	AGRB_730032	98.8	BurJV3_1288	97.3	SMD_1372	98.1	Smal_1298	96.9	SmPIER_160239	93.2	
		smlt1597	SmBFE_180152	99.4	ALOG_60163	98.4	SMBUR_110062	89.9	AGRB_810031	92.8	BurJV3_1344	91.5	SMD_1423	91.2	Smal_1354	91.1	SmPIER_170032	78.9	Unknown
		smlt1598	SmBFE_180153	99.7	ALOG_60164	99	SMBUR_110063	93.2	AGRB_810032	94.8	BurJV3_1345	96.9	SMD_1424	96.1	Smal_1355	91.5	SmPIER_170033	90.2	
		smlt1599	SmBFE_180154	98.7	ALOG_60165	99.5	SMBUR_110064	97.6	AGRB_810033	97.8	BurJV3_1346	97.8	SMD_1425	97.8	Smal1356	98.1	SmPIER_170034	93.2	
	*mac*A	smlt2642	SmBFE_210293	99.2	ALOG_110229	100	SMBUR_60037	99.2	No	No	BurJV3_2129	90.9	SMD_2312	92.1	Smal_2115	91.1	No	No	Macrolides
	*mac*B	smlt2643	SmBFE_210294	99.5	ALOG_110230	100	SMBUR_60036	99.4	No	No	BurJV3_2130	88.8	SMD_2313	88.6	Smal_2116	88.3	No	No	
	*mac*A	No	No	No	No	No	No	No	AGRB_1180029	92.2	BurJV3_2530	86.2	SMD_2659	89.4	Smal_2466		SmPIER_430055	86.9	Macrolides
	*mac*B	No	No	No	No	No	No	No	AGRB_1180030	95.2	BurJV3_2531	86.9	SMD_2660	90.8	Smal_2467		SmPIER_430056	90.3	
Fusaric acid resistance	*fua*A	smlt2796	SmBFE_230054	91.8	ALOG_130050	95.2	SMBUR_340015	93.1	AGRB_990069	89.3	No	No	SMD_2447	89.4	Smal_2253	89.4	SmPIER_400031	71.8	Fusaric acid
	*fua*B	smlt2797	SmBFE_230056	96.6	ALOG_130052	95.6	SMBUR_340013	94.9	AGRB_990071	93.2	No	No	SMD_2449	96.6	Smal_2255	92.9	SmPIER_400033	84	
	*fua*C	smlt2798	SmBFE_230057	96.2	ALOG_130053	92.3	SMBUR_340012	92.7	AGRB_990072	88.2	No	No	SMD_2450	91.6	Smal_2256	93.3	SmPIER_400034	70.1	
		smlt4662	SmBFE_420016	98.7	ALOG_210021	99	SMBUR_70016	95.1	AGRB_1040017	95.1	BurJV3_4055	96.7	SMD_4205	98.4	Smal_4009	97.1	SmPIER_570015	94.1	Fusaric acid
		smlt4663	SmBFE_420017	99.4	ALOG_210022	98.8	SMBUR_70017	97.7	AGRB_1040018	ND	BurJV3_4056	96.9	SMD_4206	97.5	Smal_4010	96.9	SmPIER_570016	94.4	
		smlt4664	SmBFE_420018	99.1	ALOG_210023	99.3	SMBUR_70018	98.2	gap	ND	BurJV3_4057	97.9	SMD_4207	98.7	Smal_4011	98.5	SmPIER_570017	95.1	
RND	*sme*V	Smlt1830	SmBFE_190100	99.7	ALOG_60541	100	SMBUR_190057	98.2	AGRB_1210020	99.7	BurJV3_1615	98.3	SMD_1764	98.7	Smal_1567	98.5	SmPIER_170257	95.4	Chloramphenicol, tetracyclines, quinolones
	*sme*W	Smlt1831	SmBFE_190101	100	ALOG_60542	100	SMBUR_190058	99.8	AGRB_1210021	99.8	BurJV3_1616	99.3	SMD_1765	99.4	Smal_1568	98.2	SmPIER_170258	94.9	
	*sme*X	Smlt1833	SmBFE_190103	99.6	ALOG_60544	99.8	SMBUR_190060	98.3	AGRB_1210023	99.4	BurJV3_1618	96.6	SMD_1767	97.9	Smal_1570	97.9	SmPIER_170260	97.5	
	*sme*Y	smlt2201	SmBFE_200142	99.5	ALOG_60932	99.7	SMBUR_150040	98.1	AGRB_1270019	97.3	BurJV3_1852	96.5	SMD_1982	95.2	Smal_1793	97.6	SmPIER_250016	90.9	Gentamycin, kanamycin, amikacin, tobramycin
	*sme*Z	smlt2202	SmBFE_200143	99.6	ALOG_60933	99.8	SMBUR_150039	99.1	AGRB_128001	99.3	BurJV3_1853	98.7	SMD_1983	98.4	Smal_1794	97.8	SmPIER_250017	93.6	
	*sme*G	smlt3170	SmBFE_280163	98.4	ALOG_130387	98.4	SMBUR_30105	96.7	AGRB_840097	97.4	BurJV3_2620	97.9	SMD_2748	96.7	Smal_2607	97.6	SmPIER_440125	92.5	Unknown
	*sme*H	smlt3171	SmBFE_280164	99.9	ALOG_130388	99.8	SMBUR_30104	99.2	AGRB_840098	99.1	BurJV3_2621	99.2	SMD_2749	98.9	Smal_2608	99.1	SmPIER_440126	98.2	
	*sme*M	smlt3788	SmBFE_330109	99.7	ALOG_150040	99.7	SMBUR_40119	95.4	AGRB_1370036	95.4	BurJV3_3235	94	SMD_3392	95.9	Smal_3204	92.4	SmPIER_500171	89.7	Unknown
	*sme*N	smlt3787	SmBFE_330108	98.7	ALOG_150039	99.8	SMBUR_40118	99	AGRB_1370035	98.9	BurJV3_3234	98.9	SMD_3391	98.9	Smal_3203	98.8	SmPIER_500170	97.8	
	*sme*O	smlt3925	SmBFE_370065	100	ALOG_160103	99.7	SMBUR_90057	98.1	AGRB_1440005	99.2	BurJV3_3376	97.3	SMD_3528	97.9	Smal_3340	77.3	SmPIER_500305	75.7	Nalidixic acid, doxycycline, amikacin, gentamycin, erythromycin, leucomycin
	*sme*P	smlt3924	SmBFE_370064	99.6	ALOG_160102	99.7	SMBUR_90056	99	AGRB_1440004	99.5	BurJV3_3375	98.6	SMD_3527	98.7	Smal_3339	91.3	SmPIER_500304	91.5	
	*tol*C	smlt3928	SmBFE_370068	100	ALOG_160106	100	SMBUR_90060	97.4	AGRB_1440008	99.1	BurJV3_3379	97.8	SMD_3531	97.8	Smal_3343	95.8	SmPIER_500308	93.1	
	*sme*D	smlt4072	SmBFE_370200	99	ALOG_160246	99.8	SMBUR_220051	98.7	AGRB_960016	96.7	BurJV3_3509	97	SMD_3658	97.7	Smal_3468	95.7	SmPIER_520028	95.4	Tetracycline, chloramphenicol, erythromycin, quinolones
	*sme*E	smlt4071	SmBFE_370199	99.5	ALOG_160245	100	SMBUR_220052	99.5	AGRB_960017	99.4	BurJV3_3508	98.9	SMD_3657	99.1	Smal_3467	96	SmPIER_520027	94.3	
	*sme*F	smlt4070	SmBFE_370198	99.6	ALOG_160244	99.8	SMBUR_220053	96.5	AGRB_960018	96.5	BurJV3_3507	96.3	SMD_3656	98.3	Smal_3466	97	SmPIER_520026	92.8	
	*sme* I	smlt4279	SmBFE_380152	99.8	ALOG_160456	99.5	SMBUR_10145	97.8	AGRB_560010	96.9	BurJV3_3723	97.1	SMD_3873	96.4	Smal_3689	96.1	SmPIER_540060	95.4	Gentamycin, amikacin, tetracycline, minocyclin, ciprofloxacin, levofloxacin
	*sme* J	smlt4280	SmBFE_380153	99.9	ALOG_160457	99.7	SMBUR_10146	99.4	AGRB_560009	98.8	BurJV3_3724	99	SMD_3874	99.4	Smal_3690	98.6	SmPIER_540061	96	
	*sme* K	smlt4281	SmBFE_380154	99.9	ALOG_160458	99.9	SMBUR_10147	98.9	AGRB_560008	97.9	BurJV3_3725	98.7	SMD_3875	99.4	Smal_3691	97.8	SmPIER_540062	96.4	
	*sme*A	smlt4476	SmBFE_400008	98.5	ALOG_180048	98.2	No	No	AGRB_760035	96.5	BurJV3_3885	95.5	SMD_4023	94	Smal_3838	92.2	No	No	Penicillin, carbenicillin, ampicillin, cefsulodin, cefotaxime, cefoperazone, cefepime, cefpirome, amikacine, gentamycine, kanamycine, streptomycin
	*sme*B	smlt4475	SmBFE_400007	99.5	ALOG_180047	99.8	No	No	AGRB_760036	97.5	BurJV3_3884	98.3	SMD_4022	97.6	Smal_3837	98.3	No	No	
	*sme*C	smlt4474	SmBFE_400006	97.7	ALOG_180046	98.1	No	No	AGRB_760037	95.8	BurJV3_3883	94.9	SMD_4021	94.5	Smal_3836	94.7	No	No	
	*eby*A	No	No	No	No	No	SMBUR_50075		No	No	No	No	No	No	No	No	No	No	Unknown
	*eby*B	No	No	No	No	No	SMBUR_50076		No	No	No	No	No	No	No	No	No	No	
	*eby*C	No	No	No	No	No	SMBUR_50074		No	No	No	No	No	No	No	No	No	No	
		No	SmBFE_170290		No	No	No	No	AGRB_1190119	99.7	No	No	No	No	No	No	No	No	Unknown
		No	SmBFE_170289		No	No	No	No	AGRB_1190118	99	No	No	No	No	No	No	No	No	
		No	SmBFE_170292		No	No	No	No	AGRB_1190122	98.1	No	No	No	No	No	No	No	No	
	MFP	No	No	No	No	No	No	No	No	No	BurJV3_2050	92.2	No	No	Smal_2022		No	No	Unknown
	RND	No	No	No	No	No	No	No	No	No	BurJV3_2051	98.5	No	No	Smal_2023		No	No	
	OMP	No	No	No	No	No	No	No	No	No	BurJV3_2052	96.7	No	No	Smal_2024		No	No	
	RND	No	No	No	No	No	No	No	No	No	BurJV3_2172		No	No	No	No	No	No	Unknown
	MFP	No	No	No	No	No	No	No	No	No	BurJV3_2173		No	No	No	No	No	No	
	MFP *acr*A	No	No	No	No	No	No	No	AGRB_720057	83.4	No	No	No	No	Smal_920		No	No	Tetracycline, ampicillin, puromycin, nalidixic acid, rifampin, chloramphenicol
	RND *acr*B	No	No	No	No	No	No	No	AGRB_720058	97.4	No	No	No	No	Smal_921		No	No	
	*mdt*A	No	No	No	No	No	No	No	No	No	No	No	No	No	Smal_3610		SmPIER_530019	89.2	Novobiocin, deoxycholate
	*mdt*B	No	No	No	No	No	No	No	No	No	No	No	No	No	Smal_3611		SmPIER_530020	95.4	
	*mdt*C	No	No	No	No	No	No	No	No	No	No	No	No	No	Smal_3612		SmPIER_530021	94.9	
	RND	No	No	No	No	No	No	No	No	No	No	No	No	No	No	No	SmPIER_340003		Unknown
	MFP	No	No	No	No	No	No	No	No	No	No	No	No	No	No	No	SmPIER_340004		

### The SMR Family

Efflux pumps belonging to the SMR family can export lipophilic compounds used as antimicrobials, primarily quaternary ammonium compounds (QACs), as well as cationic dyes. They have also been shown to confer resistance to multiple beta-lactams, macrolides, and tetracycline. These MDR efflux pumps are usually composed of an inner membrane protein encoded by a single gene. Nevertheless, some SMR efflux pumps require the coexpression of two separate SMR genes to constitute a paired SMR efflux system (Bay et al. 2008). Three genes encoding SMR efflux pumps are present in the nine strains (table 5). Among them, two are homologous to *sug*E from *E. coli* with translated amino acid sequence identity of 53% and 56%, respectively, over 99% of the protein sequence. The SugE efflux pump confers resistance to antiseptic compounds (table 5), but no antibiotics are known to constitute its substrate. The third gene encoding an SMR family efflux pump was found to be homologous to *emr*E from *E. coli* with translated amino acid sequence identity of 60% over the entire protein sequence. The EmrE efflux pump has been shown to confer resistance to beta-lactams, macrolides such as ampicillin and macrolides such as erythromycin and tetracycline, as well as to a variety of QACs such as methyl viologen, tetraphenylphosphonium, benzalkonium, cetyltrimethylammonium bromide (CTAB), cetylpyridinium chloride, and dyes, such as ethidium bromide, acriflavin/proflavin, crystal violet, pyronine and safranine.

### The MFS

The MFS constitutes the largest family of transporter proteins. Among the 17 described families belonging to the MFS, 2 of them, the 12-Transmembrane (TM) Drug/H+ Antiporter 1 (DHA-1) family and the 14-TM DHA-2 family are involved in multidrug resistance. Each of these two families encompasses numerous efflux pumps having different substrate specificities and conferring resistance against different antibiotics and other compounds. The 12-TM DHA-1 and 14-TM DHA-2 efflux pumps are usually composed of a single inner-membrane protein encoded by a single gene. However, in Gram-negative bacteria, these MFS encoding genes can be associated with genes encoding members of the MFP family that mediate the drug transport across the outer membrane of the bacteria. In some case, MFP proteins and their respective transport proteins can interact with members of the OMF protein family, which are outer membrane proteins enabling the substrate transport across the outer membrane of the Gram-negative bacteria. Thus, MFS class-like MDR efflux pumps can be encoded from 1, 2, or 3 distinct genes (Fluman and Bibi 2009). Five MFS efflux pumps of the 12-TM DHA-1 and 14-TM DHA-2 families have been identified in all of the *S. maltophilia* genomes (table 5). Among them, three were found in all the strains. One of these three efflux pumps is a tripartite efflux pump belonging to the 14-TM DHA-2 family with its own OMF. This MDR efflux pump is homologous to EmrAB of *E. coli* with translated amino acid sequences of *emr*A and *emr*B sharing about 43% and 49% sequence identity with the *E. coli* homologs, respectively. The EmrAB efflux system is known to confer resistance to some hydrophobic antibiotics, such as nalidixic acid and thiolactomycin, to hydrophobic uncouplers, such as carbonyl cyanide m-chlorophenylhydrazone and tetrachlorosalicylanilide, and to organomercurials. A gene encoding a second inner membrane protein from the 14-TM DHA-2 efflux system family was found in all the strains. This efflux pump is homologous to MdtD, a putative MDR efflux transporter described in *E. coli* with amino acid sequence identity of 49%; however, the substrates of this efflux pump are still to be deciphered. The third universally found MFS putative MDR efflux pump has a single efflux protein that belongs to the 12-TM DHA-1 family. The translated amino acid sequence of the gene encoding this protein has conserved domains with the Bcr/CflA subfamily, which encompasses efflux pumps involved in the resistance to bicyclomycin, sulfathiazole, and chloramphenicol. A second gene encoding an efflux pump of the Bcr/CflA subfamily was found in the environmental strains BurA1 and RR10 belonging to the same phylogenetic group as well as in the environmental strains PierC1 and R551-3 phylogenetically more distant from the main *S. maltophilia* clusters. All of the strains except BurA1 also hold another tripartite MDR efflux system homologous to EmrAB and its associated OMF.

### The ABC Transporters Family

ABC transporters are membrane proteins responsible for the uptake and secretion of a wide range of substrates. The ABC transporter family includes polyspecific MDR efflux systems that can accommodate a variety of unrelated substrates. ABC transporters can be composed of a single inner-membrane protein encoded by a single gene or by two half-transporter proteins encoded by two distinct genes. These two proteins assemble into a heterodimeric functional unit. Like the MFS transporter family, ABC transporters can be associated with an MFP protein and sometimes with an OMF protein (Lubelski et al. 2007). Five putative MDR ABC transporters have been found among the nine genomes. Two of them are present in all of the strains. The first is a tripartite efflux pump composed of an ABC transporter protein associated with an MFP and an OMF. The genes encoding the ABC transporter protein and the MFP are homologous to *mac*A and *mac*B from *E. coli* with translated amino acid sequences identities of 40% and 58%, respectively, over at least 90% of the length of the protein sequence. In *E. coli*, MacAB has been shown to be specifically involved in resistance toward the macrolide class of antibiotics. The second MDR ABC efflux pump is composed of one gene encoding the ATPase domain, one gene encoding the permease domain of the transporter, and one gene encoding a MFP. No homologous genes encoding efflux transporters with known substrates were identified; nevertheless, conserved protein domains and BLAST results suggested the involvement of this efflux pump in MDR mechanisms. The third efflux pump present in all strains is an inner membrane ABC efflux system encoded by the *smr*A gene. This efflux system has been characterized in *S. maltophilia* and is involved in resistance to structurally unrelated compounds including fluoroquinolones, tetracyclines, doxorubicin, and multiple dyes (Al-Hamad et al. 2009). Two other bipartite efflux systems composed of one ABC transporter protein and one MFP were found among the genomes. These two efflux systems are also homologous to MacAB from *E. coli*. One of these efflux systems was found in all the strains excepted PierC1 and RR10. The translated amino acid sequence identities of the *S. maltophilia* genes with *mac*A and *mac*B were about 32% and 47%, respectively, over the whole length of the protein sequence. The second was found in the strains JV3 and D457 from the same phylogenetic cluster, as well as in RR10, PierC1, and R551-3. The translated amino acid sequence identities with *mac*A and *mac*B were 37% and 53%, respectively, over the whole length of the protein sequence.

### Fusaric Acid Resistance Efflux Pumps

A tripartite efflux pump composed of a specific fusaric acid resistance inner-membrane protein, an MFP, and an OMF encoded by three distinct genes organized in an operon structure has been described in *S. maltophilia* (Hu et al. 2012). In this survey, two tripartite fusaric acid resistance efflux systems were found. One is present in all the strains; nevertheless, this putative fusaric acid resistance efflux pump has not been characterized yet but the translated amino acid sequences of the three genes encoding this efflux system share conserved domains with fusaric acid resistance proteins. The second tripartite fusaric acid resistance efflux pump has been described in *S. maltophilia* as the FuaABC efflux system encoded by three genes previously reported in the strain K279a genome sequence (Hu et al. 2012). This efflux system has been characterized as conferring resistance against fusaric acid when overexpressed. Except for strain JV3, FuaABC efflux system genes were found in all the strains.

### The RND Family

RND efflux pumps are known to have broad substrate profiles, including antimicrobial drugs from a wide range of classes, organic solvents, and disinfectants. These tripartite efflux systems are composed of an RND inner membrane protein and two additional components, a periplasm-spanning MFP and an OMF that are needed to remove the substrates from the cell. The RND inner-membrane part of the efflux system can also be a heteromultimeric structure of two proteins encoded by two distinct genes organized as part of an operon. The MFP is usually specific to each RND protein and the genes encoding these two proteins generally constitute operons. The OMF can also be encoded in the same operon, but there tend to be fewer different OMFs than RND/MFP pairs in a genome. Nevertheless, some OMFs can associate with numerous MDR efflux pumps belonging to different families to form effective efflux pumps. In *S. maltophilia*, eight operons encoding characterized or putative RND multidrug efflux pumps have been previously described (Crossman et al. 2008). Fifteen RND efflux pumps likely to be involved in antibiotic resistance were found among all the genomes, among which seven are present in all the strains (table 5). These seven efflux pumps belong to the eight described RND efflux pumps. Among these efflux pumps, SmeDEF, SmeOP-TolCsm, and SmeVWX are tripartite efflux systems having their own OMF. The TolCsm OMF can probably associate with SmeOP and with other efflux pumps that do not have a specific OMF to constitute effective efflux pumps (Lin et al. 2014). These efflux pumps, encoded by operons of three genes, contribute to the resistance to chloramphenicol, quinolones, macrolides, and tetracycline. The efflux pump SmeIJK encoded by an operon of three genes was also found in all the strains. This efflux pump, composed of two inner-membrane proteins constituting a heteromultimeric structure and a specific MFP, contributes to the resistance to aminoglycosides, fluoroquinolones, and tetracyclines. The three other described RND efflux pumps are encoded by two genes–operons that are encoding the RND protein and the specific MFP. These pumps are SmeGH and SmeMN, which are putatively involved in multidrug resistance mechanisms but for which substrates are unknown, as well as SmeYZ that contributes to aminoglycosides resistance. Surprisingly, the tripartite RND efflux system SmeABC, characteristic of the *S. maltophilia* species, was found in all the strains excepted BurA1 and PierC1. Three bipartite efflux pumps have been identified within particular strains. One has been found only in JV3 and another in R551-3 and RR10. These two efflux pumps contain acriflavin resistance protein-conserved domains. The third one is specific to the strain PierC1 and displays sequence identity of 40% with SmeOP. Three genes encoding an RND efflux pump having the same organization as SmeIJK were found in the strains R551-3 and PierC1. These genes are homologous to the MdtABC efflux pump encoding genes from *E. coli* with 45%, 61%, and 50% translated amino acid identities over at least 90% of the protein sequences. The MdtABC efflux system was shown to confer resistance against novobiocin and deoxycholate. Three tripartite RND efflux pumps have been found only within certain strains. One has been found in the strains BurE1 and RR10, the second in the strains R551-3 and JV3, and the third is specific to the strain BurA1. The genes encoding these tripartite efflux pumps all show conserved amino acid sequences with acriflavin resistance efflux proteins from the RND family but no homologous efflux pumps with known substrates could be attributed.

### EbyCAB, a Multiresistant Environmental Strain-Specific RND Efflux Pump

**Fig. 4.—:**
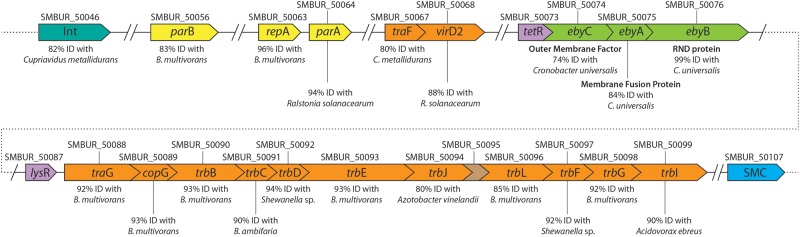
Genetic organization of the *eby*CAB genes and adjacent CDS with HGT functions located on a 63-kb genomic island of the chromosome of the strain BurA1. Locus tags are indicated on top of each CDS. CDS colored in green encodes the EbyCAB RND efflux pump specific to the BurA1 strain. CDS colored in yellow and orange are implied in mobile and extrachromosomal element functions, the orange ones belonging to the type IV secretory pathway family. CDS in pink are transcription regulators. INT, integrase; SMC, structural maintenance of chromosome protein.

As mentioned above, an RND efflux pump specific to the MDR environmental strain BurA1 was identified. In addition, this strain does not possess the SmeABC efflux pump (table 5). As this strain has the most significant resistance profile of the study, special attention was paid to the description of this efflux pump, which may have a role in resistance to antibiotics in place of SmeABC. The products of the three genes named *eby*A (SMBUR50075) encoding the MFP unit, *eby*B (SMBUR50076) encoding the RND protein, and *eby*C (SMBUR50074) encoding the OMF constitute the tripartite RND efflux pump EbyCAB. The *eby*CAB genes are organized in an operon-like structure and are preceded by a transcriptional regulator of the TetR family (SMBUR50073) (fig. 4). The *eby*CAB genes share protein sequence identity of 72.2–99.6% with an RND operon found in the *Cronobacter* and *Xanthomonas* genera from the γ-proteobacteria class, the RND protein (EbyB) having the best similarity between the three genera (99.2% identity, on average). Interestingly, only three *Cronobacter* strains (two *C. universalis* strains and one *C. muytjensii* strain) and two very closely related *Xanthomonas* strains possess this level of similarity. In fact, other strains of these two genera share no significant similarity or possess a level of similarity much lower than what is seen with these five strains (supplementary fig. S1, Supplementary Material online). Given these first observations, it seems that the *eby*CAB operon was acquired recently by horizontal transfer, at least three times independently, for each of these bacterial genera. In addition to these protein sequences exhibiting a very high similarity, protein sequences from five clinical *P. aeruginosa* strains show identity percentages with EbyB sequence of 81.9%, whereas the other RND sequences show identity percentages lower than 70%. A phylogenetic study of the EbyB protein sequence, including most of the best BLAST hits, confirms that the operon *ebyCAB* was probably transferred several times (supplementary fig. S1, Supplementary Material online).

This *eby*CAB operon is located on a genomic island of 63.3 kb identified by codon usage bias and other characteristics like the presence of an integrase similar to a phage integrase (fig. 4). Transposon and plasmid transfer genes encoding proteins from the Tra and Trb families and *par*A, *par*B and *rep*A genes, which are implicated in the maintenance and replication of mobile genetic elements, were also found in this genomic island. Most of these genes shared translated amino acid sequence identity of more than 70% over all the protein sequences belonging to the genera *Burkholderia*, *Ralstonia,* and *Acidovorax* from the ß-proteobacteria subclass. Moreover, the average of the CAI (Codon Adaptation Index) values of the whole genomic island (i.e., 57 genes) was significantly lower (*P* < 0.05; Student test) than those of the 57 upstream or downstream genes. These results suggest that the genomic structure is very mosaic which is typical of most ICE (integrative and replicative element), as defined by Burrus et al. (2002). ICE sequences are mobile elements, able to move both within a genome (as a transposon) and between strains through conjugation, after excision and circularization. A phylogenetic study of the integrase sequences shows that this genomic island belongs to the tn4371 ICE family (supplementary fig. S2, Supplementary Material online). Phylogenetic studies from other genes of the genomic island confirm that it belong to this ICE family (data not shown). The closest evolutionary ICE sequences (82.3% identity between the integrase; supplementary fig. S2, Supplementary Material online) were initially described in two soil ß-proteobacteria species (*Cupriavidus metallidurans* CH4 and *Burkholderia gladioli* BSR3). Surprisingly, the genomic islands in *S. maltophila* BurA1, *C. metallidurans* CH4 and *B. gladioli* BSR3 show strong synteny and similarity between all the ICE sequences, except for the *eby*CAB operon and some immediate flanking genes. In the two ß-proteobacteria strains, the efflux pump encoding genes have been replaced by a cluster of genes encoding metabolic functions related to carbon metabolism (*C. metallidurans* CH4) or to aromatic compound degradation (*B. gladioli* BSR3). Moreover, among the sequenced *Stenotrophomonas* strains, only one other strain (EPM1) possesses a similar ICE sequence (96.2% identity between the two integrase; supplementary fig. S2, Supplementary Material online) as the one we have described in BurA1, with its genomic location between genes encoding GMP synthase and a gene encoding an SMS protein, but without the operon *eby*, which is absent (supplementary fig. S2, Supplementary Material online). Although BurA1 and EPM1 are evolutionary closely related (fig. 2), the presence of tn4371 only in these two strains requires either two recent and independent HGTs, or an ancient gene transfer followed by a recent loss of this genomic island, in addition to the gain or loss of the *eby* operon (fig. 2). As the integration of an ICE is generally site specific (Toussaint et al. 2003), two independent integrations of tn4371 in the same place in the genome cannot be excluded.

Finally, we studied the genomic context of the RND operon close to *eby* in the *Xanthomonas* and *Cronobacter* strains (i.e., having more than 99% identity with EbyB). The immediate flanking genes are highly conserved (high synteny and identity) even if there are some chromosomal inversions and gene losses or gains, compared with the BurA1 strain (data not shown). More distant genes around the RND operon also correspond to an ICE structure (transposon plasmid and transfer genes). For the two *Xanthomonas* strains, the contig containing the RND operon available in GenBank is however too short to find the encoding integrase gene. As expected, a phylogenetic study of the integrase sequence from a *Cronobacter* strain (*C. universalis* NCTC9529) confirmed that it belongs to the tn4371 ICE family (supplementary fig. S2, Supplementary Material online). However, although the two ICE bearing genes encoding a near identical RND pump in *S. maltophila* BurA1 and *C. universalis* NCTC9529 belong actually to the same family, these two genomic islands are clearly evolutionarily distant (only 21.4% identity between the two integrase; supplementary fig. S2, Supplementary Material online ), which was confirmed with phylogenetic studies from the other genes of the ICE (data not shown). Surprisingly, the integrase of *C. universalis* NCTC9529 is evolutionarily close to an integrase from *C. metallodurans* CH4 (95.2% identity between the two integrase; supplementary fig. S2, Supplementary Material online), efflux pump encoding genes being replaced by genes encoding aromatic hydrocarbon degradation. The β-proteobacteria *C. metallodurans* CH4 has therefore two *tn4371* ICEs, each one very close to an ICE carrying the genes encoding a same efflux pump in at least two different strains of γ-proteobacteria (i.e., *Stenotrophomonas* and *Cronobacter*).

## Discussion

*Stenotrophomonas maltophilia* is a ubiquitous bacterium well known for its multiple antibiotic resistance phenotypes. As an emerging opportunistic pathogen, the antibiotic resistance mechanisms and the genes encoding them are well documented in a clinical context, but little is known on the genetic determinism of the antibiotic resistance in an environmental context. Thus, the aim of this study was to decipher the genetic determinants responsible for the variation of antibiotic resistance phenotypes among *S. maltophilia* strains recovered from the environment. This was made with emphasis on antibiotic resistance efflux pumps, as they are known to be widely involved in MDR (Li and Nikaido 2004). To this purpose, the entire pool of genetic determinants related to antibiotic resistance was assessed on whole-genome sequences of strains from different sampling origins and antibiotic resistance profiles after having defined their phylogenetic relationships by core genome phylogeny. At the beginning of our study, two genome sequences were available, one from the clinical MDR strain K279a (Crossman et al. 2008) and the other from the environmental reference endophytic strain R551-3 (Taghavi et al. 2008). Based on their exceptionally resistant or sensitive antibiotic resistance profiles, three environmental strains from the team collection were sequenced in this study. During our analysis, other genome sequences from clinical and environmental strains became available (Allen et al. 2012; Lira et al. 2012; Song et al. 2012; Zhu et al. 2012; Sassera et al. 2013; Zhang et al. 2013) but unfortunately their antibiotic resistance profiles were poorly documented.

In order to have the same level of data on the antibiotic resistance of the three sequenced environmental strains and the two reference strains, automated in vitro antibiotic resistance profiles were determined. Antibiotic susceptibility testing revealed that BurA1 and BurE1 showed resistances against 15 and 12 over 18 antibiotics assayed, respectively, including 8 and 7 antibiotic classes, respectively. If no standardized definition for MDR has been given within the scientific community, the definition most frequently used is “resistant to three or more antimicrobial classes” (Magiorakos et al. 2012). Thus BurA1 and BurE1, which have in common with the MDR reference strain K279a the resistance to seven and six antibiotic classes, can be considered as environmental strains with MDR phenotypes equivalent to that of MDR reference clinical strains. Clinical strains are frequently resistant toward more than three antibiotic classes including carbapenems, aminoglycosides, fluoroquinolones, cephalosporins, polymixins, tetracycline, and penicillins (Looney et al. 2009). Environmental strains with MDR phenotypes have been scarcely reported (Berg et al. 1999; Alouache et al. 2012). In soil and rhizosphere, Berg et al. (1999) described environmental isolates resistant toward an average of 8 antibiotics of 19 assayed, with 2 isolates resistant to 16 antibiotics and 2 isolates resistant to 14 antibiotics. Unfortunately, detailed antibiotic resistance profiles of each strain were not available, but overall these strains are resistant to the same antibiotic classes than BurA1 and BurE1 (i.e., carboxypenicillins, ureidopenicillins, cephalosporins monobactam, carbapenems, aminoglycosides, quinolones, and polymixins), as well as tetracycline and other classes not assayed in the present study. R551-3 showed resistances toward seven antibiotics belonging to six antibiotics classes. Thus according to the accepted definition of MDR (Magiorakos et al. 2012), it can be considered as an MDR environmental strain that shows an average level of resistance as reported by Berg et al. (1999). As BurA1 and BurE1 were detected in soil fields from various sites that were not exposed to any contaminants (Hien, personal communication), it appears that no antibiotic or anthropic selective pressures are needed to select antibiotic resistance among *S. maltophilia* strains. Alouache et al. (2012) described four MDR *S. maltophilia* isolates from anthropized aquatic environments, whereas no MDR isolates were isolated from nonanthropized water samples. These isolates were resistant toward a range of 15 and 18 antibiotics from 10 to 13 antibiotics classes including penicillin classes, carbapenems, aminoglycosides, tazobactam, and trimethoprim. This suggests that in some circumstances anthropization could favor antibiotic resistance among *S. maltophilia* isolates. However, it has to be noted that PierC1, an isolate from a heavy metal and antibiotic contaminated soil (Tamtam et al. 2011), only showed resistance toward imipenem, which is considered to be a natural resistance among all the *S. maltophilia* strains as this antibiotic is added in selective growth media intended for the isolation of *S. maltophilia* (Kerr et al. 1996). Thereby PierC1 constitutes an extremely sensitive strain, which complete the range of available antibiotic resistance profiles among environmental strains.

Such variability in antibiotic resistance profiles among strains from various origins raises the question of the genetic link between the resistance profile of those *S. maltophilia* strains and their phylogenetic relationships. Comparative analysis of the 3 genomes sequenced in this study with 11 available genome sequences of *S. maltophilia* revealed a core genome of 1,647 proteins representing approximately 35–45% of the total number of predicted protein-coding genes in any given genome. Consequently, these data suggest that there is tremendous latitude for variation in the genomic content of this species. The phylogenetic analysis did not correlate with the different origins of the strains. Indeed, clusters including environmental and clinical strains were observed. Thus, as previously reported in other ubiquitous Gram-negative species, most of the genes responsible for strain adaptations to its ecological niche and to pathogenesis and virulence are likely to be located on the accessory genome (Mathee et al. 2008; Sim et al. 2008; Grim et al. 2013). Despite of a lack of information on the resistance profiles of many strains, the phylogenetic analysis suggested that no distinction between resistant and sensitive strains was possible on the basis of their phylogenetic position. As expected in case of link between resistance phenotype and phylogeny, BurE1 and K279a who share a similar antibiotic resistance profile are phylogenetically closely related. They are grouped in a cluster together with the MDR strain EPM1 and the clinical strain Ab55555. This cluster might only include MDR strains but unfortunately no information on the antibiotic resistance profile of Ab55555 is available yet. Three clusters (SO28 and PML168; BurA1 and RR10; D457, JV3, and AU12-09) possibly include both antibiotic sensitive and resistant strains. Thus, on the basis of our findings it seems that at the whole genome level, antibiotic resistance is not clonal and can be acquired or lost by *S. maltophilia* strains from diverse origins with different genomic background. These observations suggest either that HGT constitutes a key mechanism for the acquisition of drug resistance determinants located on the accessory genome, or that the antibiotic resistance determinants are conserved among all *S. maltophilia* strains regardless of their antibiotic resistance profiles. A study comparing two genomes of *S. maltophilia* revealed that most antibiotic resistant genes are not associated with mobile genetic elements (Ryan et al. 2009). Thus, the second assumption might be more likely, modification of resistance phenotype essentially resulting from changes of expression and allelic variations in some conserved genes.

To determine whether the variation in the antibiotic resistance profiles of the strains was due to a different content of genetic determinants encoding resistance mechanisms, the global content of antibiotic resistance genes among nine selected genomes was compared. Between 50 and 56 genes and efflux pump operons associated with antibiotic resistance were identified in each genome. Twenty-two putative β-lactamases, 1 aminoglycoside phosphotransferase as well as 17 efflux pumps that were not, to our knowledge, previously described in *S. maltophilia* were identified during this survey, representing approximately 57% of the total pool of antibiotic resistance determinants found among the genomes. No correlation could be made between the presence or absence of a given antibiotic resistance gene and the resistance profile of an *S. maltophilia* strain. Indeed, substantially all known antibiotic resistance determinants such as *bla*L1 coding a metallo-β-lactamase responsible for the resistance toward imipenem (Walsh et al. 1994), *bla*L2 and *amp*C conferring resistance against cephalosporins and penicillins (Walsh et al. 1997; Yang et al. 2009), Sm*qnr* conferring low intrinsic resistance against quinolones (Sanchez et al. 2008; Gracia-Paez et al. 2013) and *aph*(3′)-IIc encoding an aminoglycoside phosphotransferase enzyme that increases resistance against kanamycin, neomycin, butirosin and paromomycin (Okazaki and Avison 2007), were found among all the strains regardless of their antibiotic resistance profiles or isolation origins. Comparative genomic studies of the MDR origin with other opportunistic pathogens did shed light on major differences in the antibiotic resistance gene content between resistant and sensitive strains (Fournier et al. 2006; Kumar et al. 2011), but in *S. maltophilia*, most of identified antibiotic resistance genes were present in all the genomes. Only a few antibiotic resistance genes were strain specific, including *cat*, *spg*M (McKay et al. 2003; Liaw et al. 2010), and *aac*(6′)-iz (Li et al. 2003). Nevertheless, the deficiency of these genes did not seem to impact directly the resistance phenotype of the strains as, for example, *aac*(6′)-iz was found to be absent from the genome of BurA1, but this strain is still resistant to all aminoglycosides assayed including tobramycin. Given the high number of determinants putatively involved in aminoglycoside resistance found in each genome, this could be due to functional redundancy. However among the five strains for which the detailed antibiotic resistance profile was determined, BurA1 constitutes the only strain resistant to tobramycin without carrying *aac*(6′)-iz in its genome. Thus, it could be related to a variation in the presence of MDR efflux pumps in *S. maltophilia* among which some are known to utilize aminoglycosides as substrate (Li et al. 2002; Crossman et al. 2008; Gould et al. 2013; Lin et al. 2014).

With such a small variation in the overall content of antibiotic resistance genetic determinants, the differences between the antibiotic resistance profiles could be related to the discrepancy in the content of MDR efflux pumps among the strains, the latter being heavily involved in the MDR among *S. maltophilia* (Zhang et al. 2000). As expected, numerous known and putative MDR efflux pumps were identified in each genome. The presence of many efflux pumps encoding genes in *S. maltophilia* has already been described in the first analysis of whole-genome sequence (Crossman et al. 2008). As noted in a comparison of two genomes of *S. maltophilia* (Ryan et al. 2009), the content of known and putative MDR efflux pumps of the MATE SMR, MFS, and ABC transporter families was greatly conserved between the nine genomes irrespective of their origin and resistance profiles. Two MFS, 2 ABC transporters, 1 fusaric acid resistance protein, and 1 MATE efflux pumps were not identified in all strains (table 5), but no correlation could be made between these differences in efflux pumps content and the origins and/or the resistance profiles observed. This could also be due to functional redundancy as one efflux system homologous to each of these absent efflux pump was identified in each genome. More discrepancies in the content of efflux pumps of the RND family were identified. This family of efflux pumps has been the most extensively studied regarding the impact of the efflux mechanism in *S. maltophilia* MDR strains. Eight MDR efflux pumps of the RND family referred to as Sme efflux pumps specific to *S. maltophilia* species have been described, among which six have been characterized (Alonso and Martinez 2001; Li et al. 2002; Crossman et al. 2008; Chen et al. 2011; Gould et al. 2013; Lin et al. 2014). In their study, Ryan et al. (2009) identified two additional RND efflux pumps putatively involved in MDR in the genome of the strain R551-3. As it is assumed that all strains of a given species carry the same conserved gene coding for MDR pumps in their chromosome (Alonso et al. 1999), all the Sme efflux pumps were expected to be found in every strains of *S. maltophilia*. Seven of these RND efflux pumps (SmeDEF, SmeGH, SmeIJK, SmeMN, SmeOP-TolC, SmeVWX, and SmeYZ) were identified among all the strains. Surprisingly, SmeABC could not be identified in the genomes of the strains BurA1 and PierC1. This is, to our knowledge, the first report of the absence of this *S. maltophilia*-specific efflux pump in strains belonging to this species. In addition to the two uncharacterized RND efflux pumps putatively involved in antibiotic resistance identified in the R551-3 genome (Ryan et al. 2009), five additional RND efflux pumps with putative involvement in antibiotic resistance were found among the genomes of the environmental strains only. Thus, on the basis of this analysis, the *S. maltophilia* environmental strains may carry an equal or superior amount of efflux pumps than clinical strains. Maintaining such a large number of efflux pumps in the genomes of environmental strains that are probably not faced with large doses of antibiotics supports the fact that these pumps have other roles than antibiotic resistance and maybe more related to the natural habitat of these bacteria (Piddock 2006). Some studies have emphasized the role of these efflux pumps in cell detoxification in strains associated with natural ecosystems (Piddock 2006; Poole 2007, 2008; Martinez et al. 2009) but if detoxification were the only function of these efflux systems, there would be no need to carry a large redundant number of MDR pump paralogs within the chromosome (Martinez et al. 2009). Thus it is likely that efflux can assume functions in the plant–bacteria interactions (Matilla et al. 2007; Garcia-León et al. 2014), bacterial homeostasis (Lewinson and Bibi 2001), virulence (Piddock 2006), and cell-to-cell communication (Martinez et al. 2009) justifying the conservation of such a significant number of efflux systems.

Nevertheless, the identification of a whole pool of antibiotic resistance genes included in one genome appears insufficient to the determination of the resistance profile of the corresponding strain. Indeed, a link has to be established between the presence of a gene and its functioning, especially in terms of regulation processes and variation of the expression of this gene. Many studies on the role of genetic determinants in the antibiotic resistance phenotypes showed a strong correlation between the overexpression of these determinants and the resulting MDR phenotypes (Akova et al. 1991; Alonso and Martinez 2001; Li et al. 2002; Liaw et al. 2010; Chen et al. 2011). Moreover, mutations inducing allelic variation in an antibiotic resistance gene could also modulate the resistance level conferred by a gene. In *S. maltophilia,* amino acid sequence divergences of the L1 and L2 ß-lactamases have been shown (Brooke 2012). Changes in amino acid residues of the L1 ß-lactamase were reported to alter its activity (Avison et al. 2001). Nevertheless, the different allelic variation of the Sm*qnr* gene does not seem to have a significant impact on the quinolone resistance among clinical strains (Gordon and Wareham 2010).

One of the RND efflux pumps named EbyCAB drew particular attention. This efflux system was found on a genomic island in the genome of the environmental MDR strain BurA1 sequenced in this study. The fact that the species-specific pump SmeABC was not found in the genome of BurA1 and that the strain-specific pump EbyCAB was suggests that it was very likely acquired through HGT and that this pump could have an impact on the resistance profile of this MDR strain. Interestingly, the *eby*CAB genes share high protein sequence identity (up to 99.6%) with an RND operon found in the *Cronobacter* and *Xanthomonas* genera from the γ-proteobacteria class. Unfortunately, these homologous RND efflux pumps have not been characterized yet. Although human opportunistic pathogens have been described in two of these bacterial genera (*Stenotrophomonas* and *Cronobacter*), all the strains carrying the efflux pump EbyCAB were isolated from soil or plant. Both the spread of genes encoding this pump and the selection pressures favoring their maintenance in the genome have probably an environmental (not clinical) origin. Thus, further characterization of antibiotic and natural compounds as inducers and substrates of EbyCAB is needed and is currently under investigation with the aim to understand the ecological function of EbyCAB as well as its role in the antibiotic resistance of the strain BurA1. The horizontal transfer of a functional copper resistance efflux system from *S. maltophilia* to a *Xanthomonas* strain was also described by Ryan et al. (2007). Moreover, genomic studies conducted on *S. maltophilia* reported the presence of efflux pumps involved in resistance to metals on RGPs probably acquired by HGT (Rocco et al. 2009). In *S. maltophilia,* HGT of an efflux pump involved in cadmium resistance from a phylogenetically distant Gram-positive bacterium has also been mentioned (Alonso et al. 2000), but no MDR efflux pumps horizontally acquired have been characterized yet. HGT of MDR efflux pumps located on plasmids was reported among Enterobacteriaceae, in particular efflux pumps involved in the resistance to quinolones (Deng et al. 2011) and MDR efflux system (Hansen et al. 2007). The transfer of tetracycline resistance efflux pumps presumed to be originating from *Salmonella* was also described in *Shigella* (Hartman et al. 2003) and *Acinetobacter baumanii* (Ribera et al. 2003). Here, we report, for the first time in *S. maltophilia*, an MDR efflux pump encoded by genes located in a genomic island. This genomic island has the typical structure of an ICE, able to move both in the genome and between strains by conjugation, that is, to spread rapidly in the bacterial community (Burrus et al. 2002). These genomic islands allow fast dissemination of genes involved in specific ecological functions, such as the degradation of recalcitrant organic molecules, or the resistance to different solvents or antibiotics (Toussaint et al. 2003). Surprisingly, even if the same *eby*CAB genes are shared by two ICEs in at least two bacterial genera (i.e., *Stenotrophomonas* and *Cronobacter*), these ICEs are not the same, showing the very high plasticity of these genomic islands capable of exchanging between them their gene content.

To conclude, this study showed that environmental strains and clinical ones shared similar number of resistance determinants. However, some environmental strains carried more efflux pumps than clinical ones. This confirms the findings of Ryan et al. (2009) concerning the potential broader resistance spectrum of environmental strains of *S. maltophilia*. Although allelic variations and changes of expression are probably responsible for most of the commonly encountered MDR phenotype in *S. maltophilia*, from both clinical and environmental origins, we have shown in this study that some genetic determinants involved in MDR phenotype can also be acquired by HGT. Then, the presence in the environment of MDR resistant strains and the presence of MDR efflux pump on mobile genetic elements raise questions about their potential dissemination at hospital settings and the dissemination of MDR efflux pumps between various clinical pathogens.

## Supplementary Material

Supplementary figures S1 and S2 are available at *Genome Biology and Evolution* online (http://www.gbe.oxfordjournals.org/).

Supplementary Data

## References

[evv161-B1] Akova M Bonfiglio G Livermore DM 1991 Susceptibility to beta-lactam antibiotics of mutant strains of *Xanthomonas maltophilia* with high-level and low-level constitutive expression of L1 and L2 beta-lactamases. J Med Microbiol. 35:208–213. 1941990 10.1099/00222615-35-4-208

[evv161-B2] Al-Hamad A Upton M Burnie J 2009 Molecular cloning and characterization of SmrA, a novel ABC multidrug efflux pump from *Stenotrophomonas maltophilia*. J Antimicrob Chemother. 64:731–734. 19643774 10.1093/jac/dkp271

[evv161-B3] Al-Jasser A 2006 *Stenotrophomonas maltophilia* resistant to trimethoprim-sulfamethoxazole: an increasing problem. Ann Clin Microbiol Antimicrob. 5:23. 16978420 10.1186/1476-0711-5-23 PMC1578578

[evv161-B4] Allen MJ 2012 Genome sequence of *Stenotrophomonas maltophilia* PML168, which displays Baeyer-Villiger monooxygenase activity. J Bacteriol. 194:4753–4754. 22887661 10.1128/JB.00949-12 PMC3415523

[evv161-B5] Alonso A Martinez JL 2001 Expression of multidrug efflux pump SmeDEF by clinical isolates of *Stenotrophomonas maltophilia*. Antimicrob Agents Chemother. 45:1879–1881. 11353642 10.1128/AAC.45.6.1879-1881.2001 PMC90562

[evv161-B6] Alonso A Rojo F Martinez JL 1999 Environmental and clinical isolates of *Pseudomonas aeruginosa* show pathogenic and biodegradative properties irrespective of their origin. Environ Microbiol. 1:421–430. 11207762 10.1046/j.1462-2920.1999.00052.x

[evv161-B7] Alonso A Sanchez P Martinez JL 2000 *Stenotrophomonas maltophilia* D457R contains a cluster of genes from gram-positive bacteria involved in antibiotic and heavy metal resistance. Antimicrob Agents Chemother. 44:1778–1782. 10858330 10.1128/aac.44.7.1778-1782.2000 PMC89961

[evv161-B8] Alouache S 2012 Antibiotic resistance and extended-spectrum beta-lactamases in isolated bacteria from seawater of Algiers beaches (Algeria). Microbes Environ. 27:80–86. 22095134 10.1264/jsme2.ME11266 PMC4036028

[evv161-B9] Alvarez-Ortega C Olivares J Martinez JL 2013 RND multidrug efflux pumps: what are they good for? Front Microbiol. 5:4–7. 10.3389/fmicb.2013.00007 PMC3564043 23386844

[evv161-B10] Antonioli P 2007 *Stenotrophomonas maltophilia* SeITE02, a new bacterial strain suitable for bioremediation of selenite-contaminated environmental matrices. Appl Environ Microbiol. 73:6854–6863. 17827320 10.1128/AEM.00957-07 PMC2074961

[evv161-B11] Avison MB Higgins CS von Heldreich CJ Bennett PM Walsh TR 2001 Plasmid location and molecular heterogeneity of the L1 and L2 beta-lactamase genes of *Stenotrophomonas maltophilia*. Antimicrob Agents Chemother. 45:413–419. 11158734 10.1128/AAC.45.2.413-419.2001 PMC90306

[evv161-B12] Avison MB von Heldreich CJ Higgins CS Bennett PM Walsh TR 2000 A TEM-2 beta-lactamase encoded on an active Tn1-like transposon in the genome of a clinical isolate of *Stenotrophomonas maltophilia*. J Antimicrob Chemother. 46:879–884. 11102404 10.1093/jac/46.6.879

[evv161-B13] Bay DC Rommens KL Turner RJ 2008 Small multidrug resistance proteins: a multidrug transporter family that continues to grow. Biochim Biophys Acta Biomembr. 1778:1814–1838. 10.1016/j.bbamem.2007.08.015 17942072

[evv161-B14] Benson DA 2013 GenBank. Nucleic Acids Res. 41:36–42. 10.1093/nar/gks1195 PMC3531190 23193287

[evv161-B15] Berg G Roskot N Smalla K 1999 Genotypic and phenotypic relationships between clinical and environmental isolates of *Stenotrophomas maltophilia*. J Clin Microbiol. 37:3594–3600. 10523559 10.1128/jcm.37.11.3594-3600.1999 PMC85701

[evv161-B16] Blair JM Piddock LJ 2009 Structure, function and inhibition of RND efflux pumps in Gram-negative bacteria: an update. Curr Opin Microbiol. 12:512–519. 19664953 10.1016/j.mib.2009.07.003

[evv161-B17] Bocs S Cruveiller S Vallenet D Nuel G Medigue C 2003 AMIGene: Annotation of MIcrobial Genes. Nucleic Acids Res. 31:3723–3726. 12824403 10.1093/nar/gkg590 PMC168996

[evv161-B18] Brooke J 2012 *Stenotrophomonas maltophilia*: an emerging global opportunistic pathogen. Clin Microbiol Rev. 25:2–41. 22232370 10.1128/CMR.00019-11 PMC3255966

[evv161-B19] Burrus V Pavlovic G Decaris B Guedon G 2002 Conjugative transposons: the tip of the iceberg. Mol Microbiol. 46:601–610. 12410819 10.1046/j.1365-2958.2002.03191.x

[evv161-B20] Chen CH 2011 Contribution of resistance-nodulation-division efflux pump operon *sme*U1-V-W-U2-X to multidrug resistance of *Stenotrophomonas maltophilia*. Antimicrob Agents Chemother. 55:5826–5833. 21930878 10.1128/AAC.00317-11 PMC3232770

[evv161-B22] Crossman LC 2008 The complete genome, comparative and functional analysis of *Stenotrophomonas maltophilia* reveals an organism heavily shielded by drug resistance determinants. Genome Biol. 9:R74. 18419807 10.1186/gb-2008-9-4-r74 PMC2643945

[evv161-B23] da Silva ACR 2002 Comparison of the genomes of two *Xanthomonas* pathogens with differing host specificities. Nature 417:459–463. 12024217 10.1038/417459a

[evv161-B24] Darling A Mau B Blattner F Perna N 2004 Mauve: multiple alignment of conserved genomic sequence with rearrangements. Genome Res. 14:1394–1403. 15231754 10.1101/gr.2289704 PMC442156

[evv161-B25] Deng Y 2011 Dissemination of IncFII plasmids carrying *rmt*B and *qep*A in *Escherichia coli* from pigs, farm workers and the environment. Clin Microbiol Infect. 17:1740–1745. 21375663 10.1111/j.1469-0691.2011.03472.x

[evv161-B26] Denton M Kerr KG 1998 Microbiological and clinical aspects of infection associated with *Stenotrophomonas maltophilia*. Clin Microbiol Rev. 11:57–80. 9457429 10.1128/cmr.11.1.57 PMC121376

[evv161-B27] Falagas ME Kastoris AC Vouloumanou EK Dimopoulos G 2009 Community-acquired *Stenotrophomonas maltophilia* infections: a systematic review. Eur J Clin Microbiol Infect Dis. 28:719–730. 19224257 10.1007/s10096-009-0709-5

[evv161-B28] Fluman N Bibi E 2009 Bacterial multidrug transport through the lens of the major facilitator superfamily. Biochim Biophys Acta Proteins Proteomics. 1794:738–747. 10.1016/j.bbapap.2008.11.020 19103310

[evv161-B29] Fournier PE 2006 Comparative genomics of multidrug resistance in *Acinetobacter baumannii**.* PLoS Genet. 2:62–72. 10.1371/journal.pgen.0020007 PMC1326220 16415984

[evv161-B30] Garcia-León G 2014 A function of SmeDEF, the major quinolone resistance determinant of *Stenotrophomonas maltophilia*, is the colonization of plant roots. Appl Environ Microbiol. 80:4559-4565. 24837376 10.1128/AEM.01058-14 PMC4148791

[evv161-B31] Gordon NC Wareham DW 2010 Novel variants of the Sm*qnr* family of quinolone resistance genes in clinical isolates of *Stenotrophomonas maltophilia*. J Antimicrob Chemother. 65:483–489. 20071366 10.1093/jac/dkp476

[evv161-B32] Gould VC Okazaki A Avison MB 2013 Coordinate hyperproduction of *Sme*Z and *Sme*JK efflux pumps extends drug resistance in *Stenotrophomonas maltophilia*. Antimicrob Agents Chemother. 57:655–657. 23147729 10.1128/AAC.01020-12 PMC3535947

[evv161-B33] Gracia-Paez JI 2013 Sm*qnr* variants in clinical isolates of *Stenotrophomonas maltophilia* in Brazil. Rev Inst Med Trop Sao Paulo. 55:417–420. 24213195 10.1590/S0036-46652013000600008 PMC4105090

[evv161-B34] Grim CJ 2013 Pan-genome analysis of the emerging foodborne pathogen *Cronobacter* spp. suggests a species-level bidirectional divergence driven by niche adaptation. BMC Genomics 14:366. 23724777 10.1186/1471-2164-14-366 PMC3680222

[evv161-B35] Hancock REW 1998 Resistance mechanisms in *Pseudomonas aeruginosa* and other nonfermentative gram-negative bacteria. Clin Infect Dis. 27:S93–S99. 9710677 10.1086/514909

[evv161-B36] Hansen LH Jensen LB Sorensen HI Sorensen SJ 2007 Substrate specificity of the OqxAB multidrug resistance pump in *Escherichia coli* and selected enteric bacteria. J Antimicrob Chemother. 60:145–147. 17526501 10.1093/jac/dkm167

[evv161-B37] Hartman AB Essiet II Isenbarger DW Lindler LE 2003 Epidemiology of tetracycline resistance determinants in *Shigella* spp. and enteroinvasive *Escherichia coli*: characterization and dissemination of *tet*(A)-1. J Clin Microbiol. 41:1023–1032. 12624025 10.1128/JCM.41.3.1023-1032.2003 PMC150258

[evv161-B38] Hu LF 2011 *Stenotrophomonas maltophilia* resistance to trimethoprim/sulfamethoxazole mediated by acquisition of *sul* and *dfr*A genes in a plasmid-mediated class 1 integron. Int J Antimicrob Agents. 37:230–234. 21296557 10.1016/j.ijantimicag.2010.10.025

[evv161-B39] Hu RM Liao ST Huang CC Huang YW Yang TC 2012 An inducible fusaric acid tripartite efflux pump contributes to the fusaric acid resistance in *Stenotrophomonas maltophilia*. PLoS One 7:e51053. 23236431 10.1371/journal.pone.0051053 PMC3517613

[evv161-B40] Huang YW Liou RS Lin YT Huang HH Yang TC 2014 A linkage between SmeIJK efflux pump, cell envelope integrity, and σE-mediated envelope stress response in *Stenotrophomonas maltophilia*. PLoS One 9:e111784. 25390933 10.1371/journal.pone.0111784 PMC4229105

[evv161-B41] Hunter S 2009 InterPro: the integrative protein signature database. Nucleic Acids Res. 37: D211–D215. 18940856 10.1093/nar/gkn785 PMC2686546

[evv161-B42] Kerr KG 1996 A new selective differential medium for isolation of *Stenotrophomonas maltophilia*. Eur J Clin Microbiol Infect Dis. 15:607–610. 8874082 10.1007/BF01709373

[evv161-B43] Kumar V 2011 Comparative genomics of *Klebsiella pneumoniae* strains with different antibiotic resistance profiles. Antimicrob Agents Chemother. 55:4267–4276. 21746949 10.1128/AAC.00052-11 PMC3165360

[evv161-B44] Kuroda T Tsuchiya T 2009 Multidrug efflux transporters in the MATE family. Biochim Biophys Acta Proteins Proteomics. 1794:763–768. 10.1016/j.bbapap.2008.11.012 19100867

[evv161-B45] Lewinson O Bibi E 2001 Evidence for simultaneous binding of dissimilar substrates by the *Escherichia coli* multidrug transporter MdfA. Biochemistry 40:12612–12618. 11601985 10.1021/bi011040y

[evv161-B46] Li H Durbin R 2009 Fast and accurate short read alignment with Burrows-Wheeler transform. Bioinformatics 25:1754–1760. 19451168 10.1093/bioinformatics/btp324 PMC2705234

[evv161-B47] Li XZ Nikaido H 2004 Efflux-mediated drug resistance in bacteria. Drugs 64:159–204. 14717618 10.2165/00003495-200464020-00004

[evv161-B48] Li XZ Nikaido H 2009 Efflux-mediated drug resistance in bacteria an update. Drugs 69:1555–1623. 19678712 10.2165/11317030-000000000-00000 PMC2847397

[evv161-B49] Li XZ Zhang L McKay GA Poole K 2003 Role of the acetyltransferase AAC(6')-Iz modifying enzyme in aminoglycoside resistance in *Stenotrophomonas maltophilia*. J Antimicrob Chemother. 51:803-811. 12654758 10.1093/jac/dkg148

[evv161-B50] Li XZ Zhang L Poole K 2002 SmeC, an outer membrane multidrug efflux protein of *Stenotrophomonas maltophilia*. Antimicrob Agents Chemother. 46:333–343. 11796339 10.1128/AAC.46.2.333-343.2002 PMC127032

[evv161-B51] Liaw SJ Lee YL Hsueh PR 2010 Multidrug resistance in clinical isolates of *Stenotrophomonas maltophilia*: roles of integrons, efflux pumps, phosphoglucomutase (SpgM), and melanin and biofilm formation. Int J Antimicrob Agents. 35:126–130. 19926255 10.1016/j.ijantimicag.2009.09.015

[evv161-B52] Lin CW Huang YW Hu RM Yang TC 2014 SmeOP-TolCSm efflux pump contributes to the multidrug resistance of *Stenotrophomonas maltophilia*. Antimicrob Agents Chemother. 58:2405–2408. 24395237 10.1128/AAC.01974-13 PMC4023765

[evv161-B53] Lira F 2012 Whole-genome sequence of *Stenotrophomonas maltophilia* D457, a clinical isolate and a model strain. J Bacteriol. 194:3563–3564. 22689246 10.1128/JB.00602-12 PMC3434719

[evv161-B54] Looney WJ Narita M Muhlemann K 2009 *Stenotrophomonas maltophilia*: an emerging opportunist human pathogen. Lancet Infect Dis. 9:312–323. 19393961 10.1016/S1473-3099(09)70083-0

[evv161-B55] Lubelski J Konings WN Driessen AJM 2007 Distribution and physiology of ABC-Type transporters contributing to multidrug resistance in bacteria. Microbiol Mol Biol Rev. 71:463–476. 17804667 10.1128/MMBR.00001-07 PMC2168643

[evv161-B56] Magiorakos AP 2012 Multidrug-resistant, extensively drug-resistant and pandrug-resistant bacteria: an international expert proposal for interim standard definitions for acquired resistance. Clin Microbiol. Infect. 18:268–281. 21793988 10.1111/j.1469-0691.2011.03570.x

[evv161-B58] Martinez JL 2009 Functional role of bacterial multidrug efflux pumps in microbial natural ecosystems. FEMS Microbiol Rev. 33:430–449. 19207745 10.1111/j.1574-6976.2008.00157.x

[evv161-B59] Mathee K 2008 Dynamics of *Pseudomonas aeruginosa* genome evolution. Proc Natl Acad Sci U S A. 105:3100–3105. 18287045 10.1073/pnas.0711982105 PMC2268591

[evv161-B60] Matilla MA Espinosa-Urgel M Rodriguez-Herva JJ Ramos JL Ramos-Gonzalez MI 2007 Genomic analysis reveals the major driving forces of bacterial life in the rhizosphere. Genome Biol. 8:R179. 17784941 10.1186/gb-2007-8-9-r179 PMC2375017

[evv161-B61] McKay GA Woods DE MacDonald KL Poole K 2003 Role of phosphoglucomutase of *Stenotrophomonas maltophilia* in lipopolysaccharide biosynthesis, virulence, and antibiotic resistance. Infect Immun. 71:3068–3075. 12761084 10.1128/IAI.71.6.3068-3075.2003 PMC155759

[evv161-B62] Mett H Rosta S Schacher B Frei R 1988 Outer-membrane permeability and beta-lactamase content in *Pseudomonas maltophilia* clinical isolates and laboratory mutants. Rev Infect Dis. 10:765–769. 3263685 10.1093/clinids/10.4.765

[evv161-B64] Nikaido H Pages JM 2012 Broad-specificity efflux pumps and their role in multidrug resistance of Gram-negative bacteria. FEMS Microbiol Rev. 36:340–363. 21707670 10.1111/j.1574-6976.2011.00290.x PMC3546547

[evv161-B65] Okazaki A Avison MB 2007 Aph(3′)-IIc, an aminoglycoside resistance determinant from *Stenotrophomonas maltophilia*. Antimicrob Agents Chemother. 51:359–360. 17088477 10.1128/AAC.00795-06 PMC1797691

[evv161-B67] Pages D 2008 Heavy metal tolerance in *Stenotrophomonas maltophilia*. PLoS One 3:e1539. 18253487 10.1371/journal.pone.0001539 PMC2212715

[evv161-B68] Piddock LJV 2006 Multidrug-resistance efflux pumps—not just for resistance. Nat Rev Microbiol. 4:629–636. 16845433 10.1038/nrmicro1464

[evv161-B69] Pinot C 2011 Identification of *Stenotrophomonas maltophilia* strains isolated from environmental and clinical samples: a rapid and efficient procedure. J Appl Microbiol. 111:1185–1193. 21819497 10.1111/j.1365-2672.2011.05120.x

[evv161-B70] Pitcher DG Saunders NA Owen RJ 1989 Rapid extraction of bacterial genomic DNA with guanidium thiocyanate. Lett Appl Microbiol. 8:151–156.

[evv161-B71] Poole K 2007 Efflux pumps as antimicrobial resistance mechanisms. Ann Med. 39:162–176. 17457715 10.1080/07853890701195262

[evv161-B72] Poole K 2008 Bacterial multidrug efflux pumps serve other functions. Microbe 3:179–185.

[evv161-B73] Quevillon E 2005 InterProScan: protein domains identifier. Nucleic Acids Res. 33:116–120. 10.1093/nar/gki442 PMC1160203 15980438

[evv161-B74] Ribera A Roca I Ruiz J Gibert I Vila J 2003 Partial characterization of a transposon containing the *tet*(A) determinant in a clinical isolate of *Acinetobacter baumannii*. J Antimicrob Chemother. 52:477–480. 12888597 10.1093/jac/dkg344

[evv161-B75] Rissman A 2009 Reordering contigs of draft genomes using the Mauve aligner. Bioinformatics 25:2071–2073. 19515959 10.1093/bioinformatics/btp356 PMC2723005

[evv161-B76] Rocco F De Gregorio E Colonna B Di Nocera PP 2009 *Stenotrophomonas maltophilia* genomes: a start-up comparison. Int J Med Microbiol. 299:535–546. 19574092 10.1016/j.ijmm.2009.05.004

[evv161-B77] Ryan RP 2007 An acquired efflux system is responsible for copper resistance in *Xanthomonas* strain IG-8 isolated from China. FEMS Microbiol Lett. 268:40–46. 17263848 10.1111/j.1574-6968.2006.00592.x

[evv161-B78] Ryan RP 2009 The versatility and adaptation of bacteria from the genus *Stenotrophomonas*. Nat Rev Microbiol. 7:514–525. 19528958 10.1038/nrmicro2163

[evv161-B79] Sanchez MB Hernandez A Rodriguez-Martinez JM Martinez-Martinez L Martinez JL 2008 Predictive analysis of transmissible quinolone resistance indicates *Stenotrophomonas maltophilia* as a potential source of a novel family of Qnr determinants. BMC Microbiol. 8:148. 18793450 10.1186/1471-2180-8-148 PMC2556341

[evv161-B80] Sassera D 2013 Draft genome sequence of *Stenotrophomonas maltophilia* strain EPM1, found in association with a culture of the human parasite *Giardia duodenalis*. Genome Announc. 1:e0018213. 23599297 10.1128/genomeA.00182-13 PMC3630408

[evv161-B81] Sievers F 2011 Fast, scalable generation of high-quality protein multiple sequence alignments using Clustal Omega. Mol Syst Biol. 7:539. 21988835 10.1038/msb.2011.75 PMC3261699

[evv161-B82] Sim SH 2008 The core and accessory genomes of *Burkholderia pseudomallei*: implications for human melioidosis. PLoS Pathog. 4:e1000178. 18927621 10.1371/journal.ppat.1000178 PMC2564834

[evv161-B83] Song SP 2012 Genome sequence of *Stenotrophomonas maltophilia* S028, an isolate harboring the AmpR-L2 resistance module. J Bacteriol. 194:6696. 23144428 10.1128/JB.01809-12 PMC3497473

[evv161-B84] Stamatakis A 2006 RAxML-VI-HPC: maximum likelihood-based phylogenetic analyses with thousands of taxa and mixed models. Bioinformatics 22:2688–2690. 16928733 10.1093/bioinformatics/btl446

[evv161-B85] Taghavi S 2008 Genome survey and characterization of endophytic bacteria exhibiting a beneficial effect on growth and development of poplar trees. Appl Environ Microbiol. 75:748–757. 19060168 10.1128/AEM.02239-08 PMC2632133

[evv161-B86] Talavera G Castresana J 2007 Improvement of phylogenies after removing divergent and ambiguously aligned blocks from protein sequence alignments. Syst Biol. 56:564–577. 17654362 10.1080/10635150701472164

[evv161-B87] Tamtam F 2011 Assessing the fate of antibiotic contaminants in metal contaminated soils four years after cessation of long-term waste water irrigation. Sci Total Environ. 409:540–547. 21093018 10.1016/j.scitotenv.2010.10.033

[evv161-B88] Toleman MA Bennett PM Bennett DMC Jones RN Walsh TR 2007 Global emergence of trimethoprim/sulfamethoxazole resistance in *Stenotrophomonas maltophilia* mediated by acquisition of *sul* genes. Emerg Infect Dis. 13:559–565. 17553270 10.3201/eid1304.061378 PMC2725981

[evv161-B89] Toussaint A 2003 The biphenyl- and 4-chlorobiphenyl-catabolic transposon Tn4371, a member of a new family of genomic islands related to IncP and Ti plasmids. Appl Environ Microbiol. 8:4837–4845. 12902278 10.1128/AEM.69.8.4837-4845.2003 PMC169086

[evv161-B90] Vallenet D 2006 MaGe: a microbial genome annotation system supported by synteny results. Nucleic Acids Res. 34:53–65. 16407324 10.1093/nar/gkj406 PMC1326237

[evv161-B91] Vallenet D 2009 MicroScope: a platform for microbial genome annotation and comparative genomics. Database Article ID bap021. 10.1093/database/bap021 PMC2790312 20157493

[evv161-B92] Vallenet D 2013 MicroScope—an integrated microbial resource for the curation and comparative analysis of genomic and metabolic data. Nucleic Acids Res. 41:636–647. 10.1093/nar/gks1194 PMC3531135 23193269

[evv161-B93] Walsh TR 1994 Sequence analysis of the L1 metallo beta-lactamase from *Xanthomonas maltophilia*. Biochim Biophys Acta Gene Struct Expr. 1218:199–201. 10.1016/0167-4781(94)90011-6 8018721

[evv161-B94] Walsh TR MacGowan AP Bennett PM 1997 Sequence analysis and enzyme kinetics of the L2 serine beta-lactamase from *Stenotrophomonas maltophilia*. Antimicrob Agents Chemother. 41:1460–1464. 9210666 10.1128/aac.41.7.1460 PMC163940

[evv161-B95] Yang TC Huang YW Hu RM Huang SC Lin YT 2009 AmpD(I) is involved in expression of the chromosomal L1 and L2 beta-lactamases of *Stenotrophomonas maltophilia*. Antimicrob Agents Chemother. 53:2902–2907. 19414581 10.1128/AAC.01513-08 PMC2704650

[evv161-B96] Zhang L Li XZ Poole K 2000 Multiple antibiotic resistance in *Stenotrophomonas maltophilia*: involvement of a multidrug efflux system. Antimicrob Agents Chemother. 44:287–293. 10639352 10.1128/aac.44.2.287-293.2000 PMC89673

[evv161-B97] Zhang L Morrison M Ó Cuív P Evans P Rickard CM 2013 Genome sequence of *Stenotrophomonas maltophilia* strain AU12-09, isolated from an intravascular catheter. Genome Announc. 1:e00195. 23640378 10.1128/genomeA.00195-13 PMC3642285

[evv161-B98] Zhu B 2012 Genome sequence of *Stenotrophomonas maltophilia* RR-10, isolated as an endophyte from rice root. J Bacteriol. 194:1280-1281 22328769 10.1128/JB.06702-11 PMC3294802

