# Supplementary material for: Comparative Genomics of Environmental and Clinical Stenotrophomonas maltophilia Strains with Different Antibiotic Resistance Profiles
Source: Genome Biol Evol. 2015 Sep 14;7(9):2484–505. doi: 10.1093/gbe/evv161 (PMC4607518; doi:10.1093/gbe/evv161)
Supplement: Supplementary Data [file supp_7_9_2484__index.html]

Comparative Genomics of Environmental and Clinical Stenotrophomonas maltophilia Strains with Different Antibiotic Resistance Profiles — Supplementary Data 

# Comparative Genomics of Environmental and Clinical *Stenotrophomonas maltophilia* Strains with Different Antibiotic Resistance Profiles

## Supplementary Data

files

- Supplementary Data - docx file
- Supplementary Data - docx file
