# Supplementary material for: Comparative Genomics of Environmental and Clinical Stenotrophomonas maltophilia Strains with Different Antibiotic Resistance Profiles
Source: Genome Biol Evol. 2015 Sep 14;7(9):2484–505. doi: 10.1093/gbe/evv161 (PMC4607518; doi:10.1093/gbe/evv161)
Supplement: Supplementary Data [file supp_evv161_New_Microsoft_Office_Word_Document.docx]

**Supplementary data**

**Fig. S1:** Phylogenetic tree from maximum likelihood analysis of the RND pump sequence EbyB as well as some best-BLAST hits. Bootstraps are indicated at each node. *S. maltophilia* BurA1 is highlighted in bold print.

**Fig. S2:** Phylogenetic tree from maximum likelihood analysis of integrase sequences of the *tn4371* ICE family. Bootstraps are indicated at each node. Strains highlighted in bold print share the EbyCAB encoding genes.
