# Supplementary material for: Comparative Genomics of Environmental and Clinical Stenotrophomonas maltophilia Strains with Different Antibiotic Resistance Profiles
Source: Genome Biol Evol. 2015 Sep 14;7(9):2484–505. doi: 10.1093/gbe/evv161 (PMC4607518; doi:10.1093/gbe/evv161)
Supplement: Supplementary Data [file supp_evv161_supplementary_data.docx]

*Collimonas fungivorans*, WP_ 014006145

*Variovorax paradoxus*, WP_ 021005368

*Xanthomonas arboricola*, WP_ 016902482

*Methylosarcina lacus*, WP_ 024298519

*Methylomonas sp.* FJG1, WP_ 036277251

*Methylomonas sp.* LW13, WP_ 033158236

*Methylomonas* *sp.* 11b, WP_ 026602563

*Pseudomonas cichorii* JBC1, AHF67054

*Pseudomonas viridiflava*, WP_ 025994022

*Pseudomonas viridiflava*, WP_ 029244497

*Pseudomonas syringae*, WP_ 024687292

*Pseudomonas syringae*, WP_ 024694947

*Ralstonia solanacearum*, WP_ 020829694

*Ralstonia solanacearum*, WP_003275578

*Ralstonia solanacearum*, WP_ 014616862

*Ralstonia solanacearum*, WP_ 003267501

*Oceanobacter kriegii*, WP_036536837

*Acinetobacter sp.* NIPH 2168, WP_005258454

*Pseudomonas* aeruginosa, WP*_033944424*

*Xanthomonas* *axonopodis* pv. *Malvacearum*, WP_005917670

***Stenotrophomonas maltophilia* BurA1, EbyB,** **SMBURv1_50076**

*Cronobacter muytjensii,* WP_038862367

*Cronobacter universalis*, WP_032805130

*Cronobacter universalis*, WP_038858335

0.1 substitution per site

93

99

94

100

93

100

100

66

100

100

100

99

100

58

59

100

100

98

100

62

100

**Fig. S1:** Phylogenetic tree from maximum likelihood analysis of the RND pump sequence EbyB as well as some best-BLAST hits. Bootstraps are indicated at each node. *S. maltophilia* BurA1 is highlighted in bold print.

*Cupriavidus metallidurans* CH4, ABF08348

*Burkholderia gladioli* BSR3, AEA61161

***S. maltophilia* BurA1, SMBURv1_50046**

*S. maltophilia* EPM1, AMXMv1_40083

69

67

*Polaromonas naphthalenivorans* CJ2, ABM39424

*Alicycliphilus denitrificans* BC, ADU98038

*Acidovorax sp.* JS42, ABM43095

*Acidovorax sp.* KKS102, BAJ72205

*Thioalkalivibrio sulfidophilus* HL-EbGr7, ACL72946

99

100

*Acidovorax citrulli* AAC00-1, ABM31273

*Acidovorax ebreus* TPSY, ACM34963

*Pseudomonas aeruginosa*, ACD39068

*S. maltophilia* K279a, CAQ44883

*Pseudomonas aeruginosa* PA7, ABR81390

*Bordetella petrii*, CAP42507

***Cronobacter universalis* NCTC 9529, CCK16954**

*Cupriavidus metallidurans* CH4, ABF08122

83

55

75

54

96

73

0.5 substitution per site

100

**Fig. S2**: Phylogenetic tree from maximum likelihood analysis of integrase sequences of the *tn4371* ICE family. Bootstraps are indicated at each node. Strains highlighted in bold print share the EbyCAB encoding genes.
